# Tailoring Classical Conditioning Behavior in TiO_2_ Nanowires: ZnO QDs-Based Optoelectronic Memristors for Neuromorphic Hardware

**DOI:** 10.1007/s40820-024-01338-z

**Published:** 2024-02-27

**Authors:** Wenxiao Wang, Yaqi Wang, Feifei Yin, Hongsen Niu, Young-Kee Shin, Yang Li, Eun-Seong Kim, Nam-Young Kim

**Affiliations:** 1 School of Information Science and Engineering, University of Jinan, Jinan, 250022 People’s Republic of China; 2 RFIC Centre, NDAC Centre, Kwangwoon University, Nowon-gu Seoul, 139-701 South Korea; 3 Department of Electronics Engineering, Kwangwoon University, Nowon-Gu Seoul, 139-701 South Korea; 4 School of Microelectronics, Shandong University, Jinan, 250101 People’s Republic of China; 5 Department of Molecular Medicine and Biopharmaceutical Sciences, Seoul National University, Seoul, 08826 South Korea

**Keywords:** Artificial intelligence, Classical conditioning, Neuromorphic computing, Artificial visual memory, Optoelectronic memristors, ZnO Quantum dots

## Abstract

Neuromorphic hardware equipped with associative learning capabilities presents fascinating applications in the next generation of artificial intelligence. However, research into synaptic devices exhibiting complex associative learning behaviors is still nascent. Here, an optoelectronic memristor based on Ag/TiO_2_ Nanowires: ZnO Quantum dots/FTO was proposed and constructed to emulate the biological associative learning behaviors. Effective implementation of synaptic behaviors, including long and short-term plasticity, and learning-forgetting-relearning behaviors, were achieved in the device through the application of light and electrical stimuli. Leveraging the optoelectronic co-modulated characteristics, a simulation of neuromorphic computing was conducted, resulting in a handwriting digit recognition accuracy of 88.9%. Furthermore, a 3 × 7 memristor array was constructed, confirming its application in artificial visual memory. Most importantly, complex biological associative learning behaviors were emulated by mapping the light and electrical stimuli into conditioned and unconditioned stimuli, respectively. After training through associative pairs, reflexes could be triggered solely using light stimuli. Comprehensively, under specific optoelectronic signal applications, the four features of classical conditioning, namely acquisition, extinction, recovery, and generalization, were elegantly emulated. This work provides an optoelectronic memristor with associative behavior capabilities, offering a pathway for advancing brain-machine interfaces, autonomous robots, and machine self-learning in the future.

**Supplementary Information:**

The online version contains supplementary material available at 10.1007/s40820-024-01338-z.

## Highlights

- An optoelectronic memristor with bioinspired neuromorphic behavior was proposed based on Ag/TiO_2_ Nanowires: ZnO QDs/FTO.
- The proposed device establishes superior long/short-term synaptic plasticity in response to electrical and light stimuli, and a neuromorphic computing task is effectively implemented based on its optoelectronic performance.
- The device emulated complex biological associative learning behaviors, including the four features of acquisition, extinction, restoration, and generalization.

**Supplementary Information:**

The online version contains supplementary material available at 10.1007/s40820-024-01338-z.

## Graphical abstract

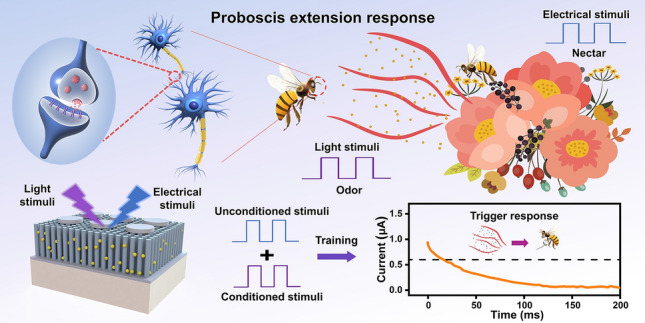

## Introduction

Artificial intelligence (AI) has made significant advancements in recent decades, thus leading to transformative changes in the means of gathering and interpreting information individually [1–6]. AI relies on computer hardware and software to simulate human intelligence and carry out various tasks. Furthermore, AI systems rely on software programming to construct and train neural networks, which are essential for performing tasks. Additionally, hardware computing units play a crucial role in processing and handling the data required for AI operations [7–9]. However, executing complex algorithmic programs is challenging using the conventional von Neumann architecture, which physically separates the memory and processing, thereby creating an inherent bottleneck in terms of computing efficiency and power consumption, and making it unsuitable for the current AI applications [10, 11]. Recently, extensive research has been conducted on neuromorphic hardware based on the human brain, which is a potential candidate for next-generation computer architectures owing to its massive parallelism, high efficiency, robust fault tolerance, and capability to integrate storage and computing [12–14]. The most challenging task in this field is mapping the biological behavior in the nervous system to the electrical behavior in devices. An artificial synapse is an emerging electronic device that can simulate the information transmission behavior between biological neurons and their intricate interconnection structure. It bridges the biological behavior and hardware characteristics, thus enabling hardware neuromorphic computing implementation [15, 16]. Currently, extensive research is being conducted to build electronic devices that can mimic essential synaptic functions. Various behaviors of the biological synapses, such as spike-timing-dependent plasticity, long-term potentiation/depression (LTP/LTD), and paired-pulse facilitation/depression (PPF/PPD), have been successfully implemented in synaptic devices [17, 18]. However, merely imitating these features is insufficient to meet the intelligent processing requirements of AI [19, 20].

An essential prerequisite for implementing synapse-like information-processing technology at the hardware level is to analyze the underlying learning and memory mechanisms observed in the biological brain [21]. The weight connection between neurons, induced by the mutual reinforcement of biological ideas and practical experience, known as associative learning behavior, forms the core of biological learning and memory [22, 23]. Classical conditioning, as a simple form of associative learning, primarily comprises conditional stimuli (CS) as well as unconditioned stimuli (US). The US can trigger an unconditioned reflex without learning, whereas the CS initially fails to trigger the reflex. When the US is repeatedly or violently accompanied by neutral stimuli, the neutral stimuli are converted to CS to produce a conditioned reflex. Classical conditioning contains four features, namely acquisition, extinction, recovery, and generalization, which correspond to the biological behaviors of information storage, elimination of old information, rememorization, and storage of new information, respectively, for an effective biomimetic synaptic device [24]. Synaptic devices with associative learning capabilities exhibit numerous captivating attributes in the context of next-generation artificial intelligence hardware. On the one hand, the hardware realization of associative learning holds the promise of enhancing the functionality of neural networks, potentially boosting the performance of machine learning algorithms, including fields such as image recognition, speech recognition, and natural language processing [25, 26]. Moreover, hardware-based associative learning facilitates the development of more autonomous machines, which can adapt and learn in dynamically changing environments without the need for pre-programming [27]. Further, synaptic hardware with associative learning capabilities demonstrates significant application potential in fields such as brain-machine interfaces and neural control [28]. However, the hardware implementation of neural networks with considerably high complexity and completeness of biological neural features is still rare [28, 29]. Among the various neurological synaptic devices, the proposal and development of a two-terminal memristor have shown promising prospects and led the way in the field of bionic electronics owing to its compact synapse-like structure and strong information transmission capability [30–33]. Several studies have demonstrated the superiority of memristors in emulating neuronal dynamics, particularly classical conditioning behavior [34, 35]. In a typical implementation, high and low-frequency/amplitude voltage pulses are used to simulate UC and CS, respectively, in classical conditioning behavior. These methods have been used to realize advanced meta-plasticity and asynchronous training and learning [36]. However, their development is limited by the inherent problems of purely electrical signals, such as crosstalk, poor sustainability, and complex circuits [37, 38]. Furthermore, the electrical signal cannot adequately realize the aforementioned four characteristics of classical conditional owing to its limited regulation methods. These challenges can be effectively solved by introducing an external stimulus orthogonal to the electric field [39–41]. Light, as a controllable, non-contact, and non-destructive stimulus, can significantly improve the tunability of biologically realistic hardware by coordinating with electrical devices. Several reports have proposed the advantages of light signals in emulating synaptic plasticity. For instance, artificial optoelectronic synapses with heterosynaptic plasticity have been effectively implemented in α-In_2_Se_3_-based devices [41]. Moreover, an application of interest-modulated human visual memories has been achieved in the ITO/Nb: SrTiO_3_ optoelectronic synapse [42]. The introduction of light stimuli greatly expands the functionality of synaptic behavior. The difference in relaxation times between light and electrical stimuli brings an inherent advantage in achieving the features of classic conditioning such as acquisition, extinction, recovery, and generalization [43, 44]. Unfortunately, few reports have employed optoelectronic synapses for the implementation of associative learning behaviors. This technology is still in its early stages and requires further research.

This study presents an optoelectronic memristor based on Ag/TiO_2_ nanowires (NWs): ZnO quantum dots (QDs)/FTO (ATZ-based device) that exhibits considerable potential for emulating classical biological conditioning along with visual memory. First, basic synaptic behaviors such as long-term and short-term synaptic plasticity (LTSP/STSP) are analyzed using electrical stimuli. Subsequently, possessing the merit of the photoconductive effect, the light-induced synaptic behaviors are investigated in detail. In addition, the resistive switching mechanisms of the ATZ-based devices are analyzed. Advanced synaptic behaviors, including short/long-term memory, learning-forgetting-relearning processes, and optoelectronic comodulated responses, are achieved by applying both electrical and light stimuli along with various parameters. An artificial neural network (ANN) is then built based on the memristor performance to verify its application in neuromorphic computing. Furthermore, a 3 × 7 optoelectronic memristor array comprising 21 ATZ-based devices is constructed to determine its potential for implementation in visual memory systems. Most importantly, light and electrical stimuli are applied to ATZ-based devices such as CS and US, respectively, to determine their ability to mimic fundamental biological conditioning, particularly for the properties of acquisition, extinction, recovery, and generalization. In summary, this study bridges the gap between the biological synaptic and optoelectronic behaviors and proposes a new method for implementing classical conditioning in hardware, thus significantly contributing to the development of AI technology.

## Experimental Section

### Materials

Zinc acetate dihydrate (C_4_H_6_O_4_Zn·2H_2_O, 99.9%), potassium hydroxide (KOH, 98%), and titanium butoxide (99.0%) were purchased from Shanghai Macklin Biochemical Technology Co. and hydrochloric acid (HCl, 37%) was purchased from Shanghai Aladdin Biochemical Technology Co., Ltd. The FTO substrates were purchased from Yingkou OPV Tech New Energy Co., Ltd.

### Preparation of the ATZ-Based Device

The ZnO QDs were synthesized using a facile solvothermal method [45, 46]. First, zinc acetate ethanol solution was prepared by dissolving 0.9790 g of zinc acetate dihydrate in 42 mL of ethanol while stirring at 60 °C. A KOH ethanol solution was prepared by dissolving 0.4859 g of KOH in 23 mL of ethanol under ultrasonic action. It was then gradually dropped into the zinc acetate ethanol solution while stirring vigorously. Subsequently, the mixed solution was maintained at 60 °C for 100 min. A transparent ZnO QD solution was obtained when the mixed solution was cooled to room temperature.

The FTO substrates were cleaned ultrasonically in acetone, ethanol, and deionized (DI) water. A facile hydrothermal method was then implemented to grow TiO_2_ NWs [47, 48]. The TiO_2_ precursor solution was obtained by mixing 10 mL of deionized (DI) water, 10 mL of HCl, and 0.4 mL of Titanium butoxide solution under ultrasonic action for 30 min. The cleaned FTO substrates were placed in a Teflon liner against the wall, and the TiO_2_ precursor solution was transferred to a Teflon-lined autoclave. Subsequently, the Teflon-lined autoclave was placed in the oven at 150 °C for 4 h. After cooling, the obtained TiO_2_ NWs film was washed thrice with DI water and dried in a vacuum oven. To incorporate the ZnO QDs, 80 μL of ZnO QD solution was dropped onto the TiO_2_ NW film surface and spin-coated at 3500 rpm for 20 s to obtain the TiO_2_ NWs: ZnO QDs film. Finally, the top Ag electrodes with a diameter of 200 μm were deposited via direct-current magnetron sputtering using a shadow mask to complete the Ag/TiO_2_ NWs: ZnO QDs/FTO-based device.

### Characterization and Measurement

The cross-sectional morphology and composition of the ATZ-based device were analyzed using a field-emission scanning electron microscope (FESEM, Regulus-800) equipped with an energy-dispersive X-ray spectroscopy (EDS) analyzer. The morphology and structure of the ZnO QDs were characterized using high-resolution transmission electron microscopy (HRTEM, FEI Tecnai F20). X-ray photoelectron spectroscopy XPS (AXIS SUPRA, Kratos) was performed to verify the chemical composition of the TiO_2_ NWs: ZnO QDs film. The photoluminescence (PL) spectra were recorded using a spectrofluorometer (PL, EH547DQ). The optical transmittance was tested by Olympus BX53M (Olympus Corp., Japan). The thickness of the Ag electrode was verified by a surface profilometer (DektakXT, Bruker, Germany). The electrical performance and synaptic functions of the ATZ-based device were analyzed at room temperature and in atmospheric environments using a Keithley 2602B source meter connected to a probe station. An ultraviolet (UV) LED light source (365 ± 10 nm) was used to test light-induced plasticity. The UV pulses were generated using a function generator (SPF80, Nanjing Shengpu instrument) and the UV light density was calibrated using a UV light meter (UV-365A, KUHNAST instrument).

## Results and Discussion

### Conceptual Design and Structural Characterizations

**Fig. 1:**
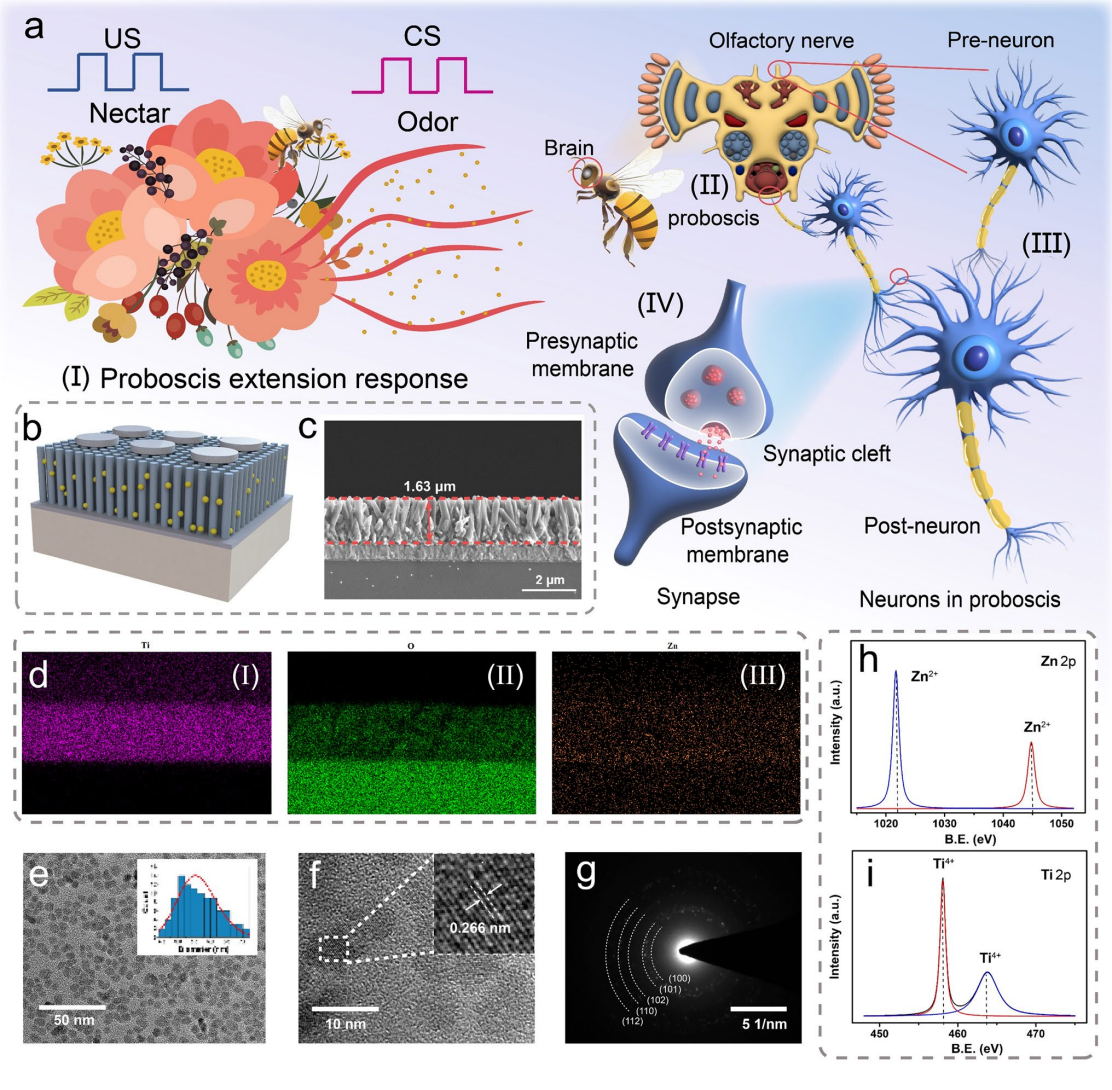
Conceptual design and structural characterizations. **a** Schematic of the proboscis extension response of a honeybee. **b** Schematic of the proposed ATZ-based device. **c** Cross-sectional SEM image of the TiO_2_ NWs: ZnO QDs/FTO/glass structure. **d** EDS elemental mapping images of (I) Ti, (II) Zn, and (III) O. **e** Low magnification TEM image of the synthesized ZnO QDs. Inset: size distribution of 100 ZnO QDs. **f** High-resolution TEM of the ZnO QDs. Inset: crystalline structure of the ZnO QDs. **g** Selected area electron diffraction (SAED) image of the ZnO QDs. XPS spectrum of the TiO_2_ NWs: ZnO QDs film: deconvoluted **h** Zn 2*p* and **i** Ti 2*p* spectra

The cranial nerves of insects exhibit highly associative learning behaviors, and a typical example is the proboscis extension response (PER) of the honeybee [34, 36]. Figure 1a presents a schematic of the PER of the honeybee. The flower nectar is the US that triggers the proboscis extension, thus indicating that the honeybee can naturally reflect the nectar, as shown in Fig. 1a-(I). The flower odor as a CS must be trained through the coordination of the olfactory and proboscis nerves before it can directly trigger the proboscis extension (Fig. 1a-(II)). PER is caused by a change in the synaptic weights between the pre and post-neurons, and time-synchronized stimuli enhance the synaptic weights of the associated neurons to adaptively associate the odor with nectar. In this process, the synapse is divided into the presynaptic membrane (to produce neurotransmitters), synaptic cleft (to transmit neurotransmitters), and postsynaptic membrane (to receive neurotransmitters), which are key components for organisms to learn and adapt to the external environment to find food or detect danger (Fig. 1a-(III) and (IV)) [32]. A two-port ATZ-based memristive device was proposed and developed in this study to emulate the synaptic behavior. Its vertical arrangement exhibits a structure similar to that of the synapses: the Ag top electrode (presynaptic membrane), TiO_2_ NWs: ZnO QDs active layer (synaptic cleft), and FTO bottom electrode (postsynaptic membrane), as shown in Fig. 1b. The as-prepared device was characterized using SEM to observe its morphology. The cross-sectional SEM image indicates that the synthesized TiO_2_ NWs grew uniformly on the FTO with a diameter of approximately 150 nm, and the thickness of the active layer was approximately 1.6 μm, as shown in Fig. 1c. Furthermore, uniformly grown nanowires were observed in the SEM image of the surface morphology (Fig. S1). A compositional analysis of the as-prepared device was performed using EDS. Figure 1d(I–III) depict the EDS mapping and elemental distribution of Ti, Zn, and O in the TiO_2_ NWs: ZnO QDs film. The transmittance of the Ag electrode was verified to ensure that the photon energy could reach the resistive active layer. As shown in Fig. S2a, the Ag/FTO structure exhibits a transmittance of 95.4% for UV light. Moreover, the thickness of the sputtered Ag electrode is detected to be ~ 29.6 nm by a surface profilometer, as shown in Fig. S2b. The photoluminescence (PL) spectra of the device were obtained at an excitation wavelength of 380 nm, which further verified the presence of ZnO QDs in the film. The peaks located at 394, 401, and 421 nm correspond to TiO_2_ NWs, whereas the peak at 548 nm can be attributed to the oxygen vacancies in the ZnO QDs, thus demonstrating the introduction of ZnO QDs, as shown in Fig. S3 [45, 49]. The morphology of the prepared ZnO QDs was characterized by TEM. Figure 1e presents the TEM image, which shows that the ZnO QDs with uniform size distribution are successfully synthesized, and the statistical size of 100 ZnO QDs in the inset demonstrates that an average size of 5.5 ± 1.5 nm can be obtained. The detailed structural information of the ZnO QDs was obtained through HRTEM (Fig. 1f). The lattice fringes confirm that the lattice spacing of the ZnO QDs is 0.266 nm, which concurs with the spacing of the wurtzite ZnO (002). The ring patterns corresponding to the (100), (101), (102), (110), and (112) planes of the ZnO QDs can be observed in the selected area electron diffraction (SAED) pattern (Fig. 1g), further demonstrating the successful synthesis of wurtzite ZnO QDs [50]. The chemical composition of the TiO_2_ NWs: ZnO QDs was analyzed using XPS (Fig. S4a). Figure 1h depicts the spectra of Zn 2*p*, where two peaks corresponding to 1022.2 and 1045.1 eV can be observed, corresponding to the Zn^2+^ of wurtzite ZnO [51]. Figure 1i depicts the XPS spectra of the deconvoluted Ti 2*p*, in which the peaks located at binding energies of 458.7 and 464.3 eV can be attributed to Ti 2*p*_3/2_ and 2*p*_1/2_, respectively, corresponding to the Ti^4+^ in TiO_2_ [52, 53]. The XPS results imply intimate contact between the TiO_2_ NWs and ZnO QDs. The O 1*s* of the TiO_2_ NWs: ZnO QDs film was exhibited in Fig. S4b, where the majority of the peak is attributed to lattice oxygen, accompanied by a small number of oxygen vacancies. To ascertain the role of ZnO in the defect centers within the device, further XPS characterization was performed on pure TiO_2_ NWs film. Figure S4c displays the Ti 2*p* spectrum of TiO_2_ NWs, showing that the Ti^4+^ peak dominates, while the area corresponding to Ti^3+^ is minimal. The O 1*s* spectrum reveals the presence of a limited quantity of oxygen defects within the TiO_2_ NWs (Fig. S4d), consequently impeding the migration of Ag ions within the bulk material [54, 55].

### Electrical Performance Evaluation

**Fig. 2:**
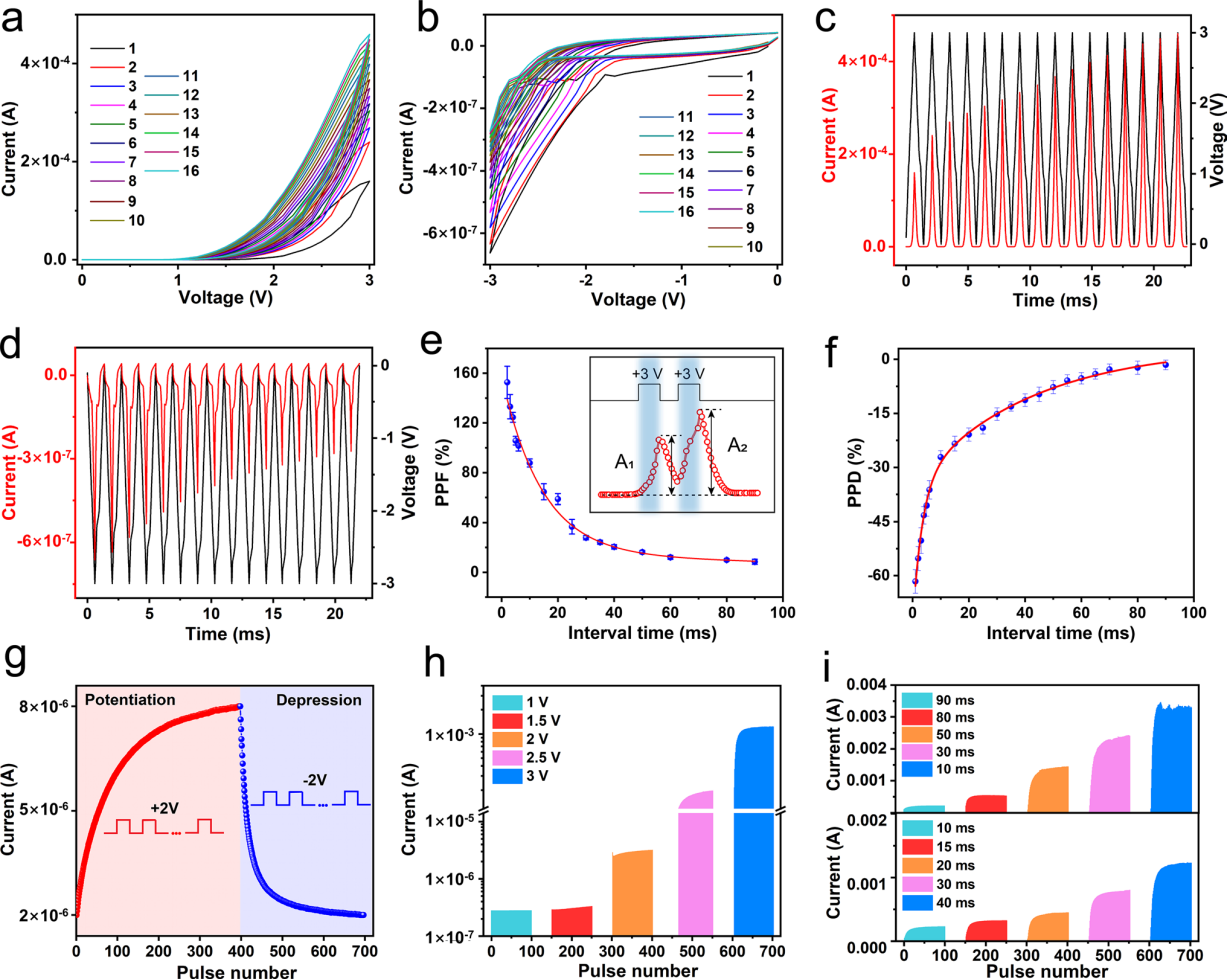
Electric-induced synaptic behavior. Consecutive *I–V* curves of the ATZ-based device for 15 cycles under **a** positive sweeping voltage (0 V → + 3 V → 0 V) and **b** negative sweeping voltage (0 V → − 3 V → 0 V). Current and voltage data vs. time of the ATZ-based device in the **c** positive and **d** negative regions. **e** PPF and **f** PPD index values (10 cycles) with respect to the interval of pulse pairs. **g** LTP/LTD properties of the ATZ-based device (+ 3 V for potentiation, − 3 V for depression). **h** Conductance variation of the ATZ-based device for different voltage pulse amplitudes. **i** Conductance variation of the ATZ-based device for different voltage durations and intervals

In artificial synapses, changes in the synaptic weight correspond to the device conductance. In this case, the potential of the ATZ-based device to emulate the synaptic behavior was verified by applying electrical signals. First, 15 cycles were consecutively applied to the top electrode. The conductance gradually increased and then decreased with the increase in the sweeping voltage, demonstrating its analogous resistive switching behavior, as shown in Fig. 2a, b. After fitting analysis of the curves, the carrier transport mechanism has been attributed to the space-charge limited current (SCLC) model resulting from the charge trapping/de-trapping behavior, which is explained in the mechanism section of the Supplementary Information. A more detailed variation in the conductance can be observed in the plot of current and voltage data vs. time. The current increases from 179.2 to 432.1 μA within 15 positive cycles, and drops from 0.65 to 0.30 μA in 15 negative cycles, as shown in Fig. 2c, d. A difference in the current values is observed between the positive and negative regions because of the contact barrier in the Ag/TiO_2_ NWs. This property is known as self-rectification and helps in high-density neural computing [56]. Notably, the synaptic behavior does not require an electrical forming process. When a forming voltage of 0 V → + 5 V → 0 V is applied to the device, the conductance of the device reaches saturation without significant conductance gradient behavior (Fig. S9). In addition, the electrical performance of pure TiO_2_ NWs-based devices is studied, as shown in Fig. S10. It can be observed that the TiO_2_ NWs-based device manifests a subtle analog resistive switching behavior, attributed to the relatively low defect concentration serving as trap centers. Subsequently, the LTSP/STSP of the ATZ-based device was analyzed in the pulse mode. A pair of voltage pulses (+ 3 V, 1 ms) was applied to the device to mimic PPF behavior. The amplitude of the current response excited by the second pulse was larger than that of the first pulse, which is consistent with biological behavior, as shown in the inset of Fig. 2e. Furthermore, the time interval between the paired pulses was regulated, and the PPF index was calculated using the following equation [57]:

1 $${\text{PPF}} = 100\% \times \frac{{{\text{A}}_{{2}} - {\text{A}}_{{1}} }}{{{\text{A}}_{{1}} }},$$

where A_1_ and A_2_ denote the currents excited by the first and second pulses, respectively. The PPF index dropped rapidly at the beginning with an increase in the interval time and decreased to approximately 8% when the interval exceeded 80 ms, and then remained stable, demonstrating a weak interaction between the first and second pulses, as shown in Fig. 2e. Two relaxation features, τ_1,_ and τ_2_, corresponding to the fast and slow decaying terms, are fitted from the PPF decay curve (10 cycles) [58]. In this case, τ_1_ and τ_2_ are fitted to 14.35 and 54.35 ms, respectively. The results obtained by fitting are relatively larger than the values observed in biological synapses [59]. Figure 2f depicts the PPD index as a function of the interval time, in which two consecutive negative pulses are applied. The PPD exhibits a decreasing trend as the interval increases, and its τ_1_ and τ_2_ are fitted to 3.39 and 41.43 ms, respectively. Figures S11 and S12 exhibit the cycle-to-cycle and device-to-device performance of the PPF/PPD properties, respectively. Furthermore, a series of voltage pulses were applied to analyze the LTSP. The current increased under the excitation of 400 consecutive voltage pulses of + 2 V (width: 50 ms, interval: 50 ms), thus indicating that the synaptic weight could be enhanced by repeated stimuli, which corresponds to the LTP, as shown in Fig. 2g. Subsequently, 300 consecutive negative pulses of − 2 V caused the current to return to its initial state, thus indicating LTD. Figure S13 depicts the LTP/LTD behavior at different pulse widths (500 μs and 500 ms). The invertal of the pulses was fixed at 50 ms. There is almost no change in the conductance of the device at 500 μs, while the conductance at 500 ms shows higher linearity but brings greater power consumption. Figure 2h, i demonstrate that the properties of synaptic weights can be controlled by electrical stimuli, that is, frequency/strength/duration-dependent synaptic plasticity [15, 60]. Larger conductance variations can be observed in the case of higher amplitude, shorter interval time, and longer duration. To verify the spontaneous decay behavior of the device, after applying 400 cycles of positive voltage pulses with different amplitudes, the conductance state is read out using 0.1 V pulses. As shown in Fig. S14, it can be observed that after the excitation process, the device exhibits varying degrees of spontaneous decay behavior, and the decay trend accelerates as the amplitude of the voltage decreases. These behaviors form the foundation for multidimensional synaptic weight updates and neural computing capabilities.

### Optoelectronic Performance Evaluation

**Fig. 3:**
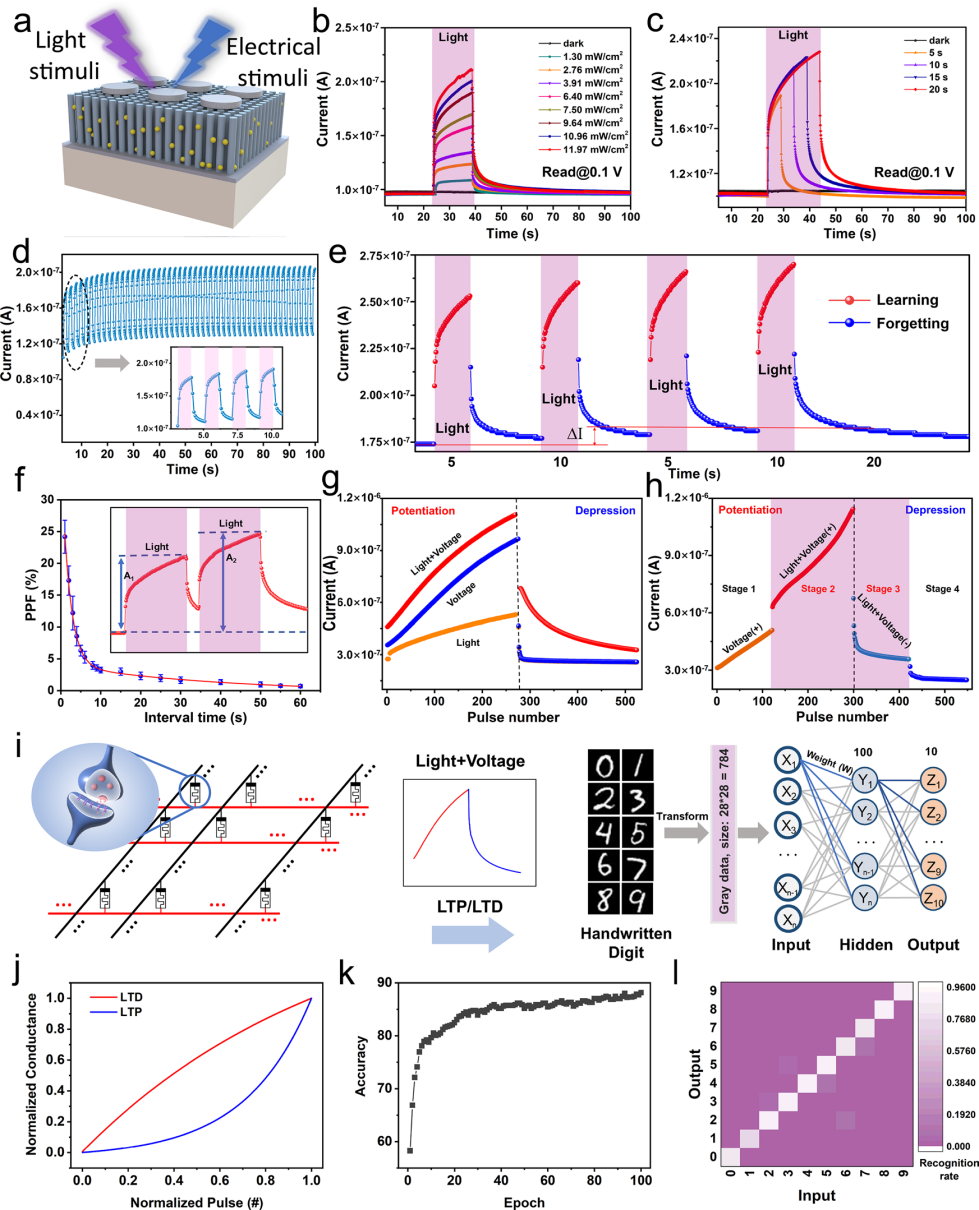
Light-induced synaptic plasticity of the ATZ-based device. **a** Schematic of the light-induced performance measurement. **b** Current responses under different light densities (dark, 1.30, 2.76, 3.91, 6.40, 7.50, 9.64, 10.96, and 11.97 mW cm^−2^). **c** Current responses under different light durations (5, 10, 15, and 20 s). **d** Current response under consecutive light pulses. Inset: local zoomed view of **d**. **e** Light-induced learning-forgetting-relearning process of four cycles. **f** Light-induced PPF behavior (10 cycles). **g** LTP/LTD performance under the excitation of light, voltage, and light plus voltage. **h** LTP/LTD performance under the synergy of light and electrical modulation. **i** Schematic of the ANN-based neural network for MNIST dataset classification using LTP/LTD (light + voltage) properties of the device. **j** Normalized conductance of the LTP/LTD vs. the normalized number of pulses. **k** Variation in accuracy during 100 training epochs. **l** Confusion matrix for recognition of the numbers from 0 to 9

Photoelectrical properties demonstrate exceptional potential in advanced neural computing and artificial visual systems. In this case, predesigned optoelectronic hybrid waveforms were applied to the ATZ-based device to analyze the light-induced synaptic plasticity (Fig. 3a). First, a light pulse with a duration of 15 s and light power density of 11.97 mW cm^−2^ was irradiated on the device, and a series of voltage pulses (0.1 V, 50 ms) were applied to simultaneously readout the conductance. When the light was turned on, the conductance of the device increased rapidly from 0.10 to 0.23 μA within 15 s. Subsequently, the conductance decayed to its initial state in approximately 60 s after turning off the light. Figure 3b depicts the conductance variations under various light densities (dark, 1.30, 2.76, 3.91, 6.40, 7.50, 9.64, 10.96, and 11.97 mW cm^−2^), where it can be observed that the response increases with density. The decay trend of the conductance between different densities after turning off the light must be analyzed carefully. The time taken for the decay from the response peak to the initial value decreases with the decrease in density, thus indicating that this type of modulation enables the ATZ-based devices to achieve a transition from STSP to LTSP. In the case of low density, the induced response decays rapidly, corresponding to short-term synaptic weight changes in response to low-density stimuli in biological synapses (STSP). Under high density, the decay trend slows down, thus indicating that the enhanced synaptic weights can be maintained for a relatively long period (LTSP). Moreover, the light response of pure TiO_2_ NWs-based devices under different light densities was investigated. As shown in Fig. S15, it can be observed that even under high light density, the decay trend of response is quite rapid. This behavior is attributed to the absence of the photogenerated electron separation effect from ZnO QDs. In the pure TiO_2_ NWs-based device, the recombination efficiency of photogenerated electron–hole pairs is relatively high, which is not conducive to the implementation of synaptic behavior. Figure 3c depicts the duration-dependent light-induced response of the ATZ-based device, in which four durations (5, 10, 15, and 20 s) were applied to the device. The ATZ-based device was transformed to a more conductive condition by using a long-duration light pulse, thus resulting in a long decay time, along with STSP-to-LTSP transitions. Additionally, the light-induced response of the ATZ-based device exhibits the frequency-dependent property, as shown in Fig. S16. The reliability of the light-induced responses was verified by applying 50 cycles of on/off light pulses to each device. The light-induced response increased slightly after each light pulse (inset of Fig. 3d) and reached saturation after 50 cycles, as shown in Fig. 3d. Overall, the light-induced response remained stable without noticeable fluctuations over 50 cycles. Furthermore, the learning and forgetting behavior of the human brain, which follows the Ebbinghaus forgetting model (Fig. S17), was mimicked by applying four cycles of on/off light pulses [61]. Light-on (5 s) represents the learning process, light-off (10 s) represents the forgetting process, and conductance represents memory strength. The first learning process increased the current from 0.18 to 0.26 μA, and after the first forgetting process, the decayed current slightly increased from the initial state, i.e., the memory strength was enhanced. The relearning process further enhanced the memory strength (decayed current after 10 s). Finally, a gap exists in the ∆I between the first and fourth decayed currents, which indicates that memory is significantly enhanced by repeated learning processes. This behavior is very similar to the human case and it demonstrates the potential of the ATZ-based device in emulating the “learning-forgetting-relearning” function. The light-induced PPF behavior was achieved by applying two consecutive light pulses at various intervals. The PPF index decays as the intervals, τ_1_ and τ_2_, are fitted to 1.62 and 36.56 s, respectively, as shown in Fig. 3f. Figure S18 depicts the cycle-to-cycle and device-to-device performance of the light-induced PPF behavior. Purely electrical pulses (1.2 V, 50 ms), purely light stimuli (6.40 mW cm^−2^), and mixed light (6.40 mW cm^−2^) and electrical stimuli (1.2 V, 50 ms) were applied to the device to increase the variation range of the response, and the current response was read out by a series of 0.1 V pluses. In these three types of modulations, the purely electrical-induced potentiation achieved a high response of 0.92 μA, whereas the purely light-induced response (reaching 0.49 μA) was significantly smaller than that of the electrical case, as shown in Fig. 3g. Furthermore, the response can be enhanced to 1.15 μA when both light and voltage are applied. In the depression process, the addition of light reduces the decay rate of the conductance. Figure 3h depicts the potentiation/depression process in four stages, where the electrical stimuli are applied from the beginning to the end, and the light stimuli are only applied at the second and third stages. Turning on the light leads to a transition from stage 1 to stage 2, wherein the response of the device increases rapidly, demonstrating the synergy between light and electrical modulation. When the light is removed from stage 3 to stage 4, the response gradually drops to the initial state, which can be attributed to the persistent photoconductivity (PPC) effect of the photosensitive active layer [37]. The synergistic effects of the light and voltage enabled the ATZ-based devices to emulate various neuroplastic dynamics.

An ANN was built and used to classify handwritten digit recognition datasets obtained from the Modified National Institute of Standards and Technology (MNIST) to analyze the potential of neuromorphic computing. During the training process, the LTP/LTD characteristics of the ATZ-based device were modeled and employed exclusively for the matrix multiplication in the neural network to update weight updates [62, 63]. It is worth noting that for the synaptic weights in hardware neural networks, the device relies on long-term plasticity over consecutive stimulations or peripheral non-volatile memory devices to maintain weights. Detailed information on the mathematical model is presented in the weight update model section of the Supplementary Information. Three types of LTP/LTD properties (pure voltage, pure light, and light plus voltage) were analyzed to obtain the nonlinearity and asymmetric nonlinearity factors (ANF). Figure S19 presents the measurement and fitting curves of LTP/LTD. The LTP/LTD properties regulated by light plus voltage stimuli exhibited the optimal nonlinearity and ANF values, as shown in Table S1. Consequently, the rule of synaptic weight update was performed in this case. A fully connected multilayer perceptron neural network, which included 784 input neurons, 100 hidden neurons, and 10 output neurons, was simulated based on the normalized LTP/LTD properties (Fig. 3j) of the ATZ-based devices, as shown in Fig. 3i. Before training, a 28 × 28 handwritten digit image was converted into grayscale values and initialized to 784 neurons in the input layer. Each neuron updated its weights under the rules of the memristor model and then passed them to the output layer after weighted summation and activation. Subsequently, a gradient descent algorithm was used to determine the minimum loss function to optimize the output results. A total of 6,000 training images and 4,000 test images were extracted from the MNIST dataset to construct the neural network. The recognition accuracy increased from 58.24 to 88.9% after 100 epochs, as shown in Fig. 3k. Moreover, the recognition accuracy within 100 epochs under three conditions is exhibited in Fig. S20. Simultaneously, a probability confusion matrix analysis of the recognition of ten numbers from 0 to 9 was performed, as shown in Fig. 3l. The probability that the number would be correctly identified was greater than 79.2%. Notably, the above results are based on the simulation of ideal memristor behavior. To reflect the impact of variability on the neural network, the effects of both cycle-to-cycle and device-to-device variations (Fig. S21) were considered in the simulation. As shown in Fig. S22, incorporating the dynamic behavior of the devices led to a decrease in recognition accuracy to 82.3%. Table S2 summarizes and compares the recognition accuracy and performance of the proposed device with previous reports.

### Visual Memory Application

**Fig. 4:**
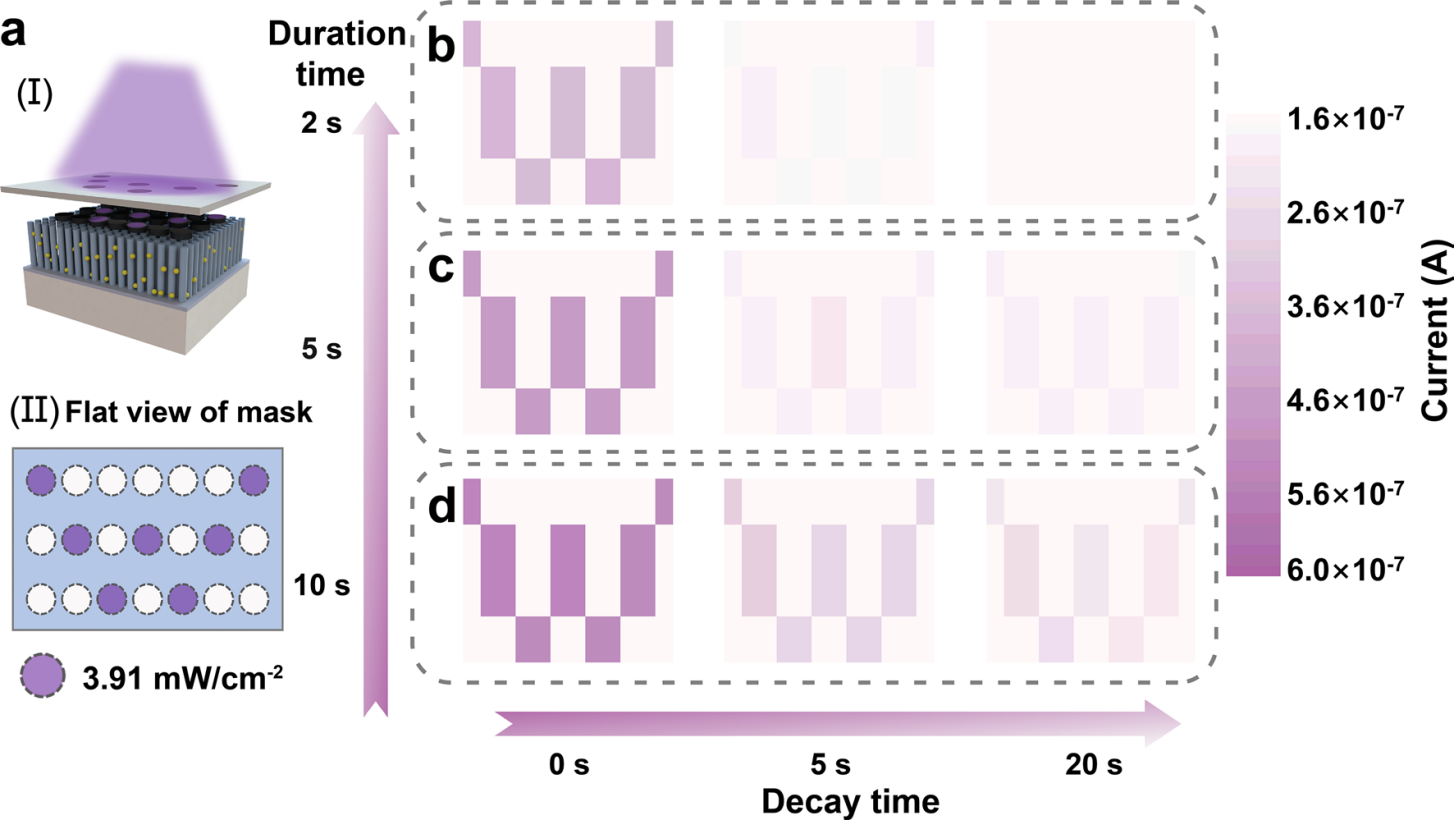
Visual memory application of the ATZ-based device. **a** Schematic diagram of light testing mapped on a 3 × 7 memristive array (I) through a “W” shaped mask, and (II) a top view of the mask. The variation in conductance-mapped pattern (letter “W”) with decay time (0, 5, and 20 s) after **b** 2 s, **c** 5 s, and **d** 10 s of light radiation

Human vision can generate a linear response corresponding to the density or duration of external light information received by the retina and it can perform short-term or long-term memory operations [43]. A 3 × 7 memristive array is constructed to mimic the human visual memory function. The optical images of the developed array can be observed in Fig. S23. Figure 4a presents the schematic of the measurement, in which the light stimuli with a light density of 3.91 mW cm^−2^ are radiated on the top electrodes of the ATZ-based device cells through a “W” shaped mask. The current is collected by testing the devices one by one by applying a probe to the exposed electrode, while the devices covered by the mask are measured in dark conditions. After 2, 5, and 10 s of excitation by the light stimuli, the variations in the conductance of each device cell with decay time are read out by voltage pulses of 0.1 V, as shown in Fig. 4b–d. A light stimulus of 2 s induces a weak conductance response; thus, the mapped letter “W” has low contrast, indicating that the intensity of visual reception of light information is insufficient for clear perception of the visual nerve, as shown in Fig. 4b. As the time decays to 5 s, the mapped letter “W” gradually blurs, until it is entirely indistinguishable after 20 s, thus indicating the forgetting of the light information. When the time of light perception increases, that is, the duration of the light stimulus increases, the light-induced response increases, and the mapped letter “W” can be perceived, as shown in Fig. 4c. After a decay of 20 s, the mapped letter is barely distinguishable instead of being completely blurred, demonstrating an extension of memory time. A high contrast mapped letter “W” can be achieved in the case of light stimuli of 10 s, and it can be resolved even after 20 s of decay, as shown in Fig. 4d. Subsequently, conductance mapping tests under pure electricity and light plus electric stimuli are implemented in the 3 × 7 memristive array. 100 electrical pulses with a pulse width of 50 ms are applied to the selected device cell, while the duration time of the light pulse is fixed at 2 s. As shown in Fig. S24, similar “W” letter mapping and decay behavior in the array can be observed. These results indicate that light information can be perceived and memorized within a short period, thus demonstrating its application potential in emulating human visual memory functions.

### Associative Learning Behavior Evaluation

**Fig. 5:**
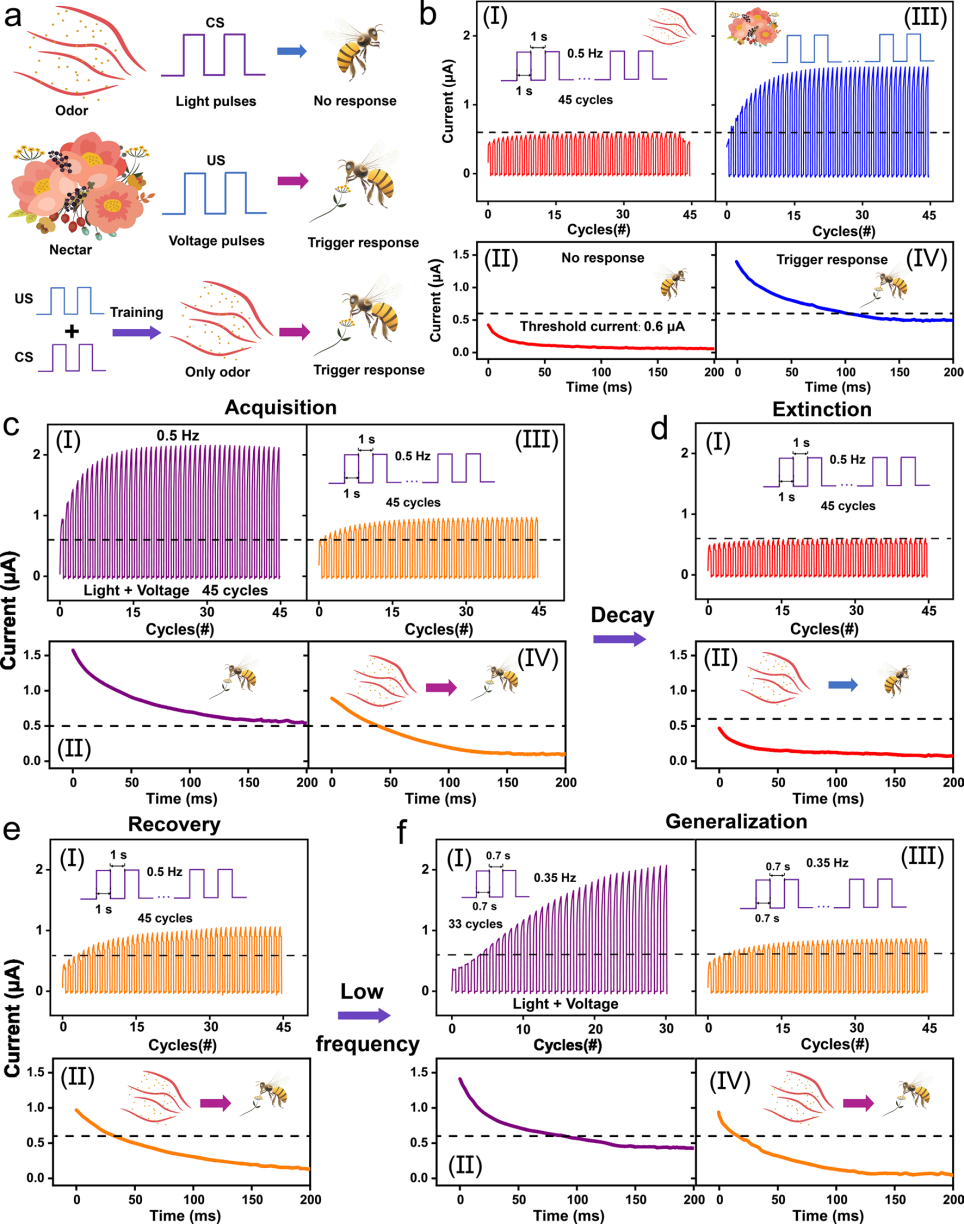
**a** Schematic of the classical PER of a honeybee. Light pulses emulate the CS (odor, no trigger response), and voltage pulses mimic the US (nectar, trigger response). **b** (I) Conductance response after excitement by CS (45 cycles of purely light pulses, 0.5 Hz) and (II) the decayed response within 200 ms after being excited by CS. (III) Conductance response after excitement by US (45 cycles of purely voltage pulses, 0.5 Hz) and (IV) the decayed response within 200 ms after being excited by US. Implement the four features in classical PER: **c** acquisition, **d** extinction, **e** recovery, and **f** generalization

Classical conditional reflex behavior, which dominates the associative learning behavior in the biological brain, has been extensively analyzed in bionic electronics research. In this study, the PER behavior of honeybees is employed as a paradigm to exhibit the associative learning behavior in the device. During the implementation, the light pulses are denoted as CS (odor), and the voltage pulses are denoted as US (nectar), as shown in Fig. 5a. The US naturally triggers proboscis extension (unconditioned response, UR), whereas the CS can induce proboscis extension only after repeated training using associative pairs of US (conditioned response, CR). During the measurement, a series of reading voltage pulses (0.1 V, 2 ms) is provided to read out the conductance response, and the threshold current is set at 0.6 μA to distinguish the occurrence of PER. The CR increases to ≈ 0.58 μA and cannot exceed the threshold even though the device is excited by consecutive CS inputs (45 cycles of purely light pulses, 0.5 Hz), thus indicating that the reflex is not triggered, as shown in Fig. 5b-(I). Subsequently, the light was turned off, and the decaying current over time was observed by applying reading voltage pulses, as shown in Fig. 5b-(II). The decaying current remained constant below the threshold (initial: 0.45 μA). Conversely, under the excitation of consecutive US (45 cycles of purely voltage pulses, 0.5 Hz), the UR gradually exceeds the threshold (reaches ≈ 1.55 μA), thus indicating that the PER is triggered (Fig. 5b-(III)). Figure 5b-(IV) depicts the decaying current over time after excitation by the US. The decaying current was initially above the threshold current corresponding to the response status; it then gradually decreased below the threshold within 100 ms, thus indicating synaptic action potential firing until the end of the reflex. Classical conditioning is a temporary neural connection established based on unconditioned reflexes and manifests four features: acquisition, extinction, recovery, and generalization. These features correspond to the storage of information, elimination of old information, rememorization, and storage of new information in the biological brain. Therefore, the acquisition feature is the basic condition required to establish a neural connection between CS and US. In this case, associative pairs comprising light stimuli (CS, 0.5 Hz) and voltage stimuli (US, 0.5 Hz) were concurrently applied to the device for acquisition. The conductance response rapidly increases above the threshold, triggering the PER, then rises to ≈ 2.19 μA after 45 training cycles, as shown in Fig. 5c-(I). The sum of the individual response (≈ 2.13 μA) is slightly lower than the case induced by the associative pairs (≈ 2.19 μA), which can be attributed to the saturated excited states in the active layer. Figure 5c-(II) depicts the decaying current after removing the associative pairs; evidently, the PER can be maintained until 200 ms without relaxing below the threshold. Immediately after training with the associative pairs, the triggering behavior of the PER was investigated using only the CS. The CR reaches ≈ 0.92 μA after 45 cycles of light stimuli, which is sufficiently large to trigger PER, thus indicating the establishment of a connection between the US and CS (acquisition), as shown in Fig. 5c-(III). Figure 5c-(IV) shows that the decaying current is initially above the threshold (≈ 0.89 μA) and then gradually decreases below the threshold at ≈ 45 ms, representing the relaxation of the PER. The training results of the associative pairs demonstrate that only the CS could trigger the response; however, this neural connection is not permanent. Extinction can dominate the disappearance of the conditioning response. After 10 min, 45 cycles of the light stimulus (CS, 0.5 Hz) were applied to each device. The CR below the threshold in Fig. 5d-(I) indicates that the CS alone failed to trigger the PER after 10 s of decay time, and the initial decaying current (≈ 0.48 μA) after excitation also remained constant below the threshold (extinction). However, the initial decaying current after training is slightly larger than that of the untrained case (Fig. 5b-(II), ≈ 0.45 μA) and below the threshold, thus demonstrating the weak neural connection between the US and CS. Additionally, synaptic plasticity enables previously extinguished responses to be activated rapidly and with large magnitudes when the same associative pairs arrive, which is the feature of recovery. Figure 5e-(I) depicts the conductance response after training using the same associative pairs. The CR is enhanced to approximately 0.97 μA, and the initial decaying current is excited to 0.95 μA (Fig. 5e-(II)), both of which are larger than the previous acquisition (conductance response: 0.92 μA, initial decaying current: 0.89 μA), thus indicating the re-strengthening of the neural connection between the US and CS (recovery). Under generalization, similar stimuli can also trigger the same CR after forming a neural connection under specific associative pairs. Low-frequency associative pairs comprising light stimuli (CS, 0.35 Hz) and voltage stimuli (US, 0.35 Hz) were considered as similar stimuli and applied simultaneously to the device after a recovery period. The conductance response gradually increased above the threshold, and the PER was effectively triggered even if the triggering process was slow, as shown in Fig. 5f-(I). In Fig. 5f-(II), the decaying current initially decreases from approximately 1.41 μA and continues to decay below the threshold at 88 ms. Similar stimuli elicit a weaker response and demonstrate a faster decay tendency compared with the high-frequency training. The CR was tested after training with similar stimuli, and it gradually increased to approximately 0.81 μA, as shown in Fig. 5f-(III); this proves that the CS can trigger PER. Subsequent measurements of decaying currents (Fig. 5f-(II)) also verified that similar stimuli can trigger the PER and establish neural connections. However, for generalization, the differentiated stimulus produced a lower response than the previous stimulus, as can be observed from the low CR and fast decay trend. In summary, the PER of the honeybee was emulated in the ATZ-based device, and its four key features, i.e., acquisition, extinction, recovery, and generalization, were successfully implemented.

## Conclusions

This study presented an optoelectronic memristor based on Ag/TiO_2_ NWs: ZnO QDs/FTO, which was designed to accomplish the classical hardware conditioning reflex. Under electrical stimuli, the device exhibited a hysteresis *I–V* curve with a gradual change in conductance, which could be attributed to charge trapping/de-trapping. Moreover, basic synaptic behaviors, such as LTP/LTD and PPF/PPD, were effectively achieved. Furthermore, several advanced synaptic plasticities, including the transition from STSP to LTSP, light-induced LTP/LTD, and learning-forgetting-relearning functions were achieved under the synergy of light and electrical modulation. An ANN exhibiting a high recognition accuracy of 88.9% for the MNIST dataset after training for 100 epochs was also established using coordinated modulated performance. The potential for emulating artificial visual memory was validated by employing a 3 × 7 memristive array consisting of the proposed device. Most importantly, the PER of a honeybee was demonstrated using associative pairs of light and voltage stimuli, which functioned as conditional and unconditional stimuli, respectively. In summary, an effective biomimetic synaptic optoelectronic memristor was developed in this study, which presents considerable potential for the development of hardware, artificial intelligence, neuroprosthetics, and neurorobotics.

## Supplementary Information

Below is the link to the electronic supplementary material.

Supplementary file1 (PDF 2178 KB)

## References

[1] C. Li, D. Belkin, Y. Li, P. Yan, M. Hu et al., Efficient and self-adaptive in situ learning in multilayer memristor neural networks. Nat. Commun. **9**, 2385 (2018). 10.1038/s41467-018-04484-2 29921923 10.1038/s41467-018-04484-2 PMC6008303

[2] J. Pei, L. Deng, S. Song, M. Zhao, Y. Zhang et al., Towards artificial general intelligence with hybrid Tianjic chip architecture. Nature **572**, 106–111 (2019). 10.1038/s41586-019-1424-8 31367028 10.1038/s41586-019-1424-8

[3] C. Wan, P. Cai, M. Wang, Y. Qian, W. Huang et al., Artificial sensory memory. Adv. Mater. **32**, e1902434 (2020). 10.1002/adma.201902434 31364219 10.1002/adma.201902434

[4] C. Li, Z. Wang, M. Rao, D. Belkin, W. Song et al., Long short-term memory networks in memristor crossbar arrays. Nat. Mach. Intell. **1**, 49–57 (2019). 10.1038/s42256-018-0001-4

[5] J. Jumper, R. Evans, A. Pritzel, T. Green, M. Figurnov et al., Highly accurate protein structure prediction with AlphaFold. Nature **596**, 583–589 (2021). 10.1038/s41586-021-03819-2 34265844 10.1038/s41586-021-03819-2 PMC8371605

[6] G. Carleo, M. Troyer, Solving the quantum many-body problem with artificial neural networks. Science **355**, 602–606 (2017). 10.1126/science.aag2302 28183973 10.1126/science.aag2302

[7] Z. Wang, S. Joshi, S. Savel’ev, W. Song, R. Midya et al., Fully memristive neural networks for pattern classification with unsupervised learning. Nat. Electron. **1**, 137–145 (2018). 10.1038/s41928-018-0023-2

[8] E. Peterson, A. Lavin, Physical computing for materials acceleration platforms. Matter **5**, 3586–3596 (2022). 10.1016/j.matt.2022.09.022

[9] K. Hippalgaonkar, Q. Li, X. Wang, J.W. Fisher III., J. Kirkpatrick et al., Knowledge-integrated machine learning for materials: lessons from gameplaying and robotics. Nat. Rev. Mater. **8**, 241–260 (2023). 10.1038/s41578-022-00513-1

[10] Y. Lee, H.-L. Park, Y. Kim, T.-W. Lee, Organic electronic synapses with low energy consumption. Joule **5**, 794–810 (2021). 10.1016/j.joule.2021.01.005

[11] J.-Q. Yang, R. Wang, Y. Ren, J.-Y. Mao, Z.-P. Wang et al., Neuromorphic engineering: from biological to spike-based hardware nervous systems. Adv. Mater. **32**, e2003610 (2020). 10.1002/adma.202003610 33165986 10.1002/adma.202003610

[12] C. Eckel, J. Lenz, A. Melianas, A. Salleo, R.T. Weitz, Nanoscopic electrolyte-gated vertical organic transistors with low operation voltage and five orders of magnitude switching range for neuromorphic systems. Nano Lett. **22**, 973–978 (2022). 10.1021/acs.nanolett.1c03832 35049308 10.1021/acs.nanolett.1c03832

[13] B.J. Shastri, A.N. Tait, T. Ferreira de Lima, W.H.P. Pernice, H. Bhaskaran et al., Photonics for artificial intelligence and neuromorphic computing. Nat. Photonics **15**, 102–114 (2021). 10.1038/s41566-020-00754-y

[14] Z. Lv, Y. Wang, J. Chen, J. Wang, Y. Zhou et al., Semiconductor quantum dots for memories and neuromorphic computing systems. Chem. Rev. **120**, 3941–4006 (2020). 10.1021/acs.chemrev.9b00730 32202419 10.1021/acs.chemrev.9b00730

[15] J. Zeng, J. Zhao, T. Bu, G. Liu, Y. Qi et al., A flexible tribotronic artificial synapse with bioinspired neurosensory behavior. Nano-Micro Lett. **15**, 18 (2022). 10.1007/s40820-022-00989-0 10.1007/s40820-022-00989-0 PMC9800681 36580114

[16] S. Najmaei, A.L. Glasmann, M.A. Schroeder, W.L. Sarney, M.L. Chin et al., Advancements in materials, devices, and integration schemes for a new generation of neuromorphic computers. Mater. Today **59**, 80–106 (2022). 10.1016/j.mattod.2022.08.017

[17] S.H. Sung, T.J. Kim, H. Shin, T.H. Im, K.J. Lee, Simultaneous emulation of synaptic and intrinsic plasticity using a memristive synapse. Nat. Commun. **13**, 2811 (2022). 10.1038/s41467-022-30432-2 35589710 10.1038/s41467-022-30432-2 PMC9120471

[18] L. Wang, W. Liao, S.L. Wong, Z.G. Yu, S. Li et al., Artificial synapses based on multiterminal memtransistors for neuromorphic application. Adv. Funct. Mater. **29**, 1901106 (2019). 10.1002/adfm.201901106

[19] M. Seo, M.-H. Kang, S.-B. Jeon, H. Bae, J. Hur et al., First demonstration of a logic-process compatible junctionless ferroelectric FinFET synapse for neuromorphic applications. IEEE Electron Device Lett. **39**, 1445–1448 (2018). 10.1109/LED.2018.2852698

[20] Y.-C. Chiang, C.-C. Hung, Y.-C. Lin, Y.-C. Chiu, T. Isono et al., High-performance nonvolatile organic photonic transistor memory devices using conjugated rod-coil materials as a floating gate. Adv. Mater. **32**, e2002638 (2020). 10.1002/adma.202002638 32700349 10.1002/adma.202002638

[21] J. Hochstetter, R. Zhu, A. Loeffler, A. Diaz-Alvarez, T. Nakayama et al., Avalanches and edge-of-chaos learning in neuromorphic nanowire networks. Nat. Commun. **12**, 4008 (2021). 10.1038/s41467-021-24260-z 34188085 10.1038/s41467-021-24260-z PMC8242064

[22] W. Schultz, A. Dickinson, Neuronal coding of prediction errors. Annu. Rev. Neurosci. **23**, 473–500 (2000). 10.1146/annurev.neuro.23.1.473 10845072 10.1146/annurev.neuro.23.1.473

[23] R.A. Poldrack, J. Clark, E.J. Paré-Blagoev, D. Shohamy, J. Creso Moyano et al., Interactive memory systems in the human brain. Nature **414**, 546–550 (2001). 10.1038/35107080 11734855 10.1038/35107080

[24] H. Zhang, H. Zeng, A. Priimagi, O. Ikkala, Viewpoint: Pavlovian materials—functional biomimetics inspired by classical conditioning. Adv. Mater. **32**, 1906619 (2020). 10.1002/adma.201906619 10.1002/adma.201906619 32003096

[25] Z. Wang, C. Li, W. Song, M. Rao, D. Belkin et al., Reinforcement learning with analogue memristor arrays. Nat. Electron. **2**, 115–124 (2019). 10.1038/s41928-019-0221-6

[26] J.H. Baek, K.J. Kwak, S.J. Kim, J. Kim, J.Y. Kim et al., Two-terminal lithium-mediated artificial synapses with enhanced weight modulation for feasible hardware neural networks. Nano-Micro Lett. **15**, 69 (2023). 10.1007/s40820-023-01035-3 10.1007/s40820-023-01035-3 PMC10030746 36943534

[27] K. He, Y. Liu, J. Yu, X. Guo, M. Wang et al., Artificial neural pathway based on a memristor synapse for optically mediated motion learning. ACS Nano **16**, 9691–9700 (2022). 10.1021/acsnano.2c03100 35587990 10.1021/acsnano.2c03100

[28] J. Sun, G. Han, Z. Zeng, Y. Wang, Memristor-based neural network circuit of full-function Pavlov associative memory with time delay and variable learning rate. IEEE Trans. Cybern. **50**, 2935–2945 (2020). 10.1109/TCYB.2019.2951520 31751264 10.1109/TCYB.2019.2951520

[29] A. Rao, P. Plank, A. Wild, W. Maass, A long short-term memory for AI applications in spike-based neuromorphic hardware. Nat. Mach. Intell. **4**, 467–479 (2022). 10.1038/s42256-022-00480-w

[30] Q. Liu, S. Gao, L. Xu, W. Yue, C. Zhang et al., Nanostructured perovskites for nonvolatile memory devices. Chem. Soc. Rev. **51**, 3341–3379 (2022). 10.1039/d1cs00886b 35293907 10.1039/d1cs00886b

[31] D.B. Strukov, G.S. Snider, D.R. Stewart, R.S. Williams, The missing memristor found. Nature **453**, 80–83 (2008). 10.1038/nature06932 18451858 10.1038/nature06932

[32] S.H. Jo, T. Chang, I. Ebong, B.B. Bhadviya, P. Mazumder et al., Nanoscale memristor device as synapse in neuromorphic systems. Nano Lett. **10**, 1297–1301 (2010). 10.1021/nl904092h 20192230 10.1021/nl904092h

[33] M. Chen, M. Sun, H. Bao, Y. Hu, B. Bao, Flux–charge analysis of two-memristor-based chua’s circuit: dimensionality decreasing model for detecting extreme multistability. IEEE Trans. Ind. Electron. **67**, 2197–2206 (2020). 10.1109/TIE.2019.2907444

[34] C. Wu, T.W. Kim, T. Guo, F. Li, D.U. Lee et al., Mimicking classical conditioning based on a single flexible memristor. Adv. Mater. **29**, 1602890 (2017). 10.1002/adma.201602890 10.1002/adma.201602890 27996165

[35] S. Wang, D.W. Zhang, P. Zhou, Two-dimensional materials for synaptic electronics and neuromorphic systems. Sci. Bull. **64**, 1056–1066 (2019). 10.1016/j.scib.2019.01.016 10.1016/j.scib.2019.01.016 36659765

[36] M. Ziegler, R. Soni, T. Patelczyk, M. Ignatov, T. Bartsch et al., An electronic version of pavlov’s dog. Adv. Funct. Mater. **22**, 2744–2749 (2012). 10.1002/adfm.201200244

[37] W. Wang, S. Gao, Y. Li, W. Yue, H. Kan et al., Artificial optoelectronic synapses based on TiN_*x*_O_2–__*x*_/MoS_2_ heterojunction for neuromorphic computing and visual system. Adv. Funct. Mater. **31**, 2170247 (2021). 10.1002/adfm.202170247

[38] K. Wang, J. Chen, X. Yan, MXene Ti_3_C_2_ memristor for neuromorphic behavior and decimal arithmetic operation applications. Nano Energy **79**, 105453 (2021). 10.1016/j.nanoen.2020.105453

[39] W. Wang, S. Gao, Y. Wang, Y. Li, W. Yue et al., Advances in emerging photonic memristive and memristive-like devices. Adv. Sci. **9**, e2105577 (2022). 10.1002/advs.202105577 10.1002/advs.202105577 PMC9534950 35945187

[40] M. Spagnolo, J. Morris, S. Piacentini, M. Antesberger, F. Massa et al., Experimental photonic quantum memristor. Nat. Photonics **16**, 318–323 (2022). 10.1038/s41566-022-00973-5

[41] K. Liu, T. Zhang, B. Dang, L. Bao, L. Xu et al., An optoelectronic synapse based on α-In_2_Se_3_ with controllable temporal dynamics for multimode and multiscale reservoir computing. Nat. Electron. **5**, 761–773 (2022). 10.1038/s41928-022-00847-2

[42] S. Gao, G. Liu, H. Yang, C. Hu, Q. Chen et al., An oxide Schottky junction artificial optoelectronic synapse. ACS Nano **13**, 2634–2642 (2019). 10.1021/acsnano.9b00340 30730696 10.1021/acsnano.9b00340

[43] D. Kumar, A. Saleem, L.B. Keong, Y.H. Wang, T.-Y. Tseng, Light induced RESET phenomenon in invisible memristor for photo sensing. IEEE Electron Device Lett. **43**, 1069–1072 (2022). 10.1109/LED.2022.3172866

[44] Y. Pei, L. Yan, Z. Wu, J. Lu, J. Zhao et al., Artificial visual perception nervous system based on low-dimensional material photoelectric memristors. ACS Nano **15**, 17319–17326 (2021). 10.1021/acsnano.1c04676 34541840 10.1021/acsnano.1c04676

[45] W. Wang, Y. Li, W. Yue, S. Gao, C. Zhang et al., Study on multilevel resistive switching behavior with tunable ON/OFF ratio capability in forming-free ZnO QDs-based RRAM. IEEE Trans. Electron Devices **67**, 4884–4890 (2020). 10.1109/TED.2020.3022005

[46] T.J. Jacobsson, S. Viarbitskaya, E. Mukhtar, T. Edvinsson, A size dependent discontinuous decay rate for the exciton emission in ZnO quantum dots. Phys. Chem. Chem. Phys. **16**, 13849–13857 (2014). 10.1039/c4cp00254g 24658340 10.1039/c4cp00254g

[47] B. Liu, E.S. Aydil, Growth of oriented single-crystalline rutile TiO_2_ nanorods on transparent conducting substrates for dye-sensitized solar cells. J. Am. Chem. Soc. **131**, 3985–3990 (2009). 10.1021/ja8078972 19245201 10.1021/ja8078972

[48] Z. Pan, H. Rao, I. Mora-Seró, J. Bisquert, X. Zhong, Quantum dot-sensitized solar cells. Chem. Soc. Rev. **47**, 7659–7702 (2018). 10.1039/c8cs00431e 30209490 10.1039/c8cs00431e

[49] M. Rajabi, S. Shogh, A. Iraji zad, Defect study of TiO_2_ nanorods grown by a hydrothermal method through photoluminescence spectroscopy. J. Lumin. **157**, 235–242 (2015). 10.1016/j.jlumin.2014.08.035

[50] S. Liu, M.-Y. Li, D. Su, M. Yu, H. Kan et al., Broad-band high-sensitivity ZnO colloidal quantum dots/self-assembled Au nanoantennas heterostructures photodetectors. ACS Appl. Mater. Interfaces **10**, 32516–32525 (2018). 10.1021/acsami.8b09442 30165735 10.1021/acsami.8b09442

[51] Y. Wang, Y. Gong, L. Yang, Z. Xiong, Z. Lv et al., MXene-ZnO memristor for multimodal In-sensor computing. Adv. Funct. Mater. **31**, 2100144 (2021). 10.1002/adfm.202100144

[52] M. Xiao, K.P. Musselman, W.W. Duley, N.Y. Zhou, Resistive switching memory of TiO_2_ nanowire networks grown on Ti foil by a single hydrothermal method. Nano-Micro Lett. **9**, 15 (2016). 10.1007/s40820-016-0116-2 10.1007/s40820-016-0116-2 PMC6223795 30460312

[53] W. Liu, Y. Yun, M. Li, J. Mao, C. Li et al., Preparation of hollow ceramic photocatalytic membrane grafted with silicon-doped TiO_2_ nanorods and conversion of high-concentration NO. Chem. Eng. J. **437**, 135261 (2022). 10.1016/j.cej.2022.135261

[54] M.S. Irshad, A. Abbas, H.H. Qazi, M.H. Aziz, M. Shah et al., Role of point defects in hybrid phase TiO_2_ for resistive random-access memory (RRAM). Mater. Res. Express **6**, 076311 (2019). 10.1088/2053-1591/ab17b5

[55] P. Russo, M. Xiao, R. Liang, N.Y. Zhou, UV-induced multilevel current amplification memory effect in zinc oxide rods resistive switching devices. Adv. Funct. Mater. **28**, 1706230 (2018). 10.1002/adfm.201706230

[56] W. Wang, R. Wang, T. Shi, J. Wei, R. Cao et al., A self-rectification and quasi-linear analogue memristor for artificial neural networks. IEEE Electron Device Lett. **40**, 1407–1410 (2019). 10.1109/LED.2019.2929240

[57] J.-T. Yang, C. Ge, J.-Y. Du, H.-Y. Huang, M. He et al., Artificial synapses emulated by an electrolyte-gated tungsten-oxide transistor. Adv. Mater. (2018). 10.1002/adma.201801548 10.1002/adma.201801548 29974526

[58] S.J. Kim, T.H. Lee, J.-M. Yang, J.W. Yang, Y.J. Lee et al., Vertically aligned two-dimensional halide perovskites for reliably operable artificial synapses. Mater. Today **52**, 19–30 (2022). 10.1016/j.mattod.2021.10.035

[59] Y. Lin, J. Liu, J. Shi, T. Zeng, X. Shan et al., Nitrogen-induced ultralow power switching in flexible ZnO-based memristor for artificial synaptic learning. Appl. Phys. Lett. **118**, 103502 (2021). 10.1063/5.0036667

[60] K.C. Kwon, J.H. Baek, K. Hong, S.Y. Kim, H.W. Jang, Memristive devices based on two-dimensional transition metal chalcogenides for neuromorphic computing. Nano-Micro Lett. **14**, 58 (2022). 10.1007/s40820-021-00784-3 10.1007/s40820-021-00784-3 PMC8818077 35122527

[61] T. Chang, S.-H. Jo, W. Lu, Short-term memory to long-term memory transition in a nanoscale memristor. ACS Nano **5**, 7669–7676 (2011). 10.1021/nn202983n 21861506 10.1021/nn202983n

[62] P. Zhang, M. Xia, F. Zhuge, Y. Zhou, Z. Wang et al., Nanochannel-based transport in an interfacial memristor can emulate the analog weight modulation of synapses. Nano Lett. **19**, 4279–4286 (2019). 10.1021/acs.nanolett.9b00525 31150262 10.1021/acs.nanolett.9b00525

[63] Q. Liu, S. Gao, Y. Li, W. Yue, C. Zhang et al., HfO_2_/WO_3_ heterojunction structured memristor for high-density storage and neuromorphic computing. Adv. Mater. Technol. **8**, 2201143 (2023). 10.1002/admt.202201143

