# Supplementary material for: Tailoring Classical Conditioning Behavior in TiO2 Nanowires: ZnO QDs-Based Optoelectronic Memristors for Neuromorphic Hardware
Source: Nanomicro Lett. 2024 Feb 27;16:133. doi: 10.1007/s40820-024-01338-z (PMC10899558; doi:10.1007/s40820-024-01338-z)
Supplement: Supplementary file 1 — Supplementary file1 (PDF 2178 KB) [file 40820_2024_1338_MOESM1_ESM.pdf]

Supporting Information for

Tailoring Classical Conditioning Behavior in TiO_2 Nanowires: ZnO QDs-Based Optoelectronic Memristors for Neuromorphic Hardware

Wenxiao Wang[1,2,3,#], Yaqi Wang[4,#], Feifei Yin[1,3], Hongsen Niu[1,3], Young-Kee Shin[5], Yang Li[1,2,4,*], Eun-Seong Kim[1,3,*], and Nam-Young Kim[1,3,5,*]

[1] RFIC Centre, NDAC Centre, Kwangwoon University, 20 Kwangwoon-ro, Nowon-Gu, Seoul, 01897, South Korea

[2] School of Microelectronics, Shandong University, Jinan 250101, P. R. China

[3] Department of Electronics Engineering, Kwangwoon University, 20 Kwangwoon-ro, Nowon-Gu, Seoul, 01897, South Korea

[4] School of Information Science and Engineering, University of Jinan, Jinan 250022, P. R. China

[5] Department of Molecular Medicine and Biopharmaceutical Sciences, Seoul National University, Seoul, 08826, South Korea

#W. Wang and Y. Wang contributed equally to this study.

*Corresponding authors. E-mail: yang.li@sdu.edu.cn (Yang Li); 3037eskim@gmail.com (Eun-Seong Kim); nykm@kw.ac.kr (Nam-Young Kim)

Supplementary Figures and Tables

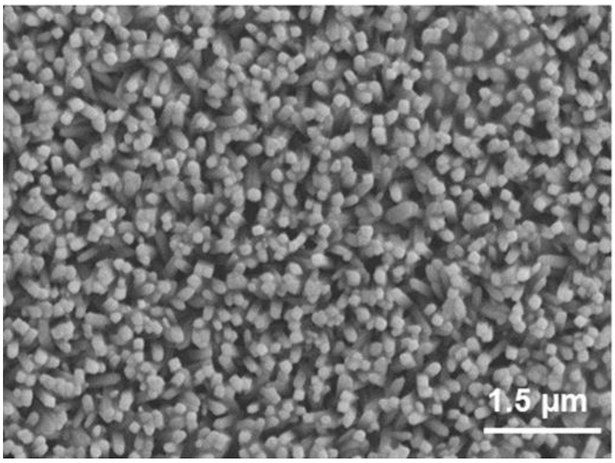

Fig. S1 SEM image of the surface morphology of the TiO_2 NWs: ZnO QDs film

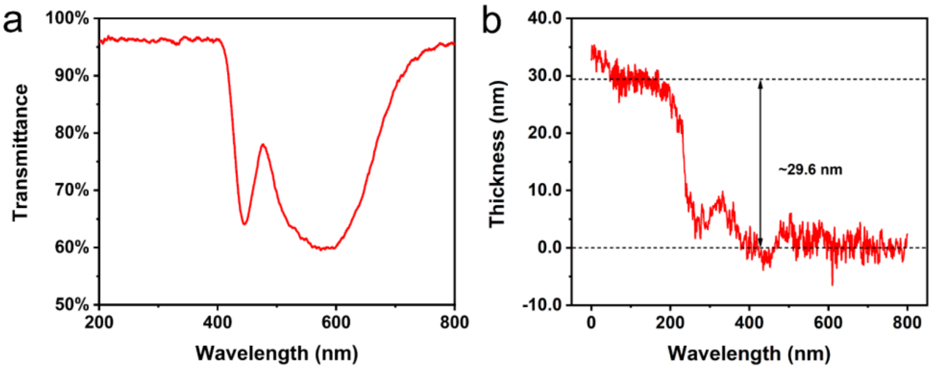

Fig. S2 a Optical transmittance spectrum of Ag electrode/FTO substrate (wavelength: 200-800 nm). **b** Thickness of the Ag electrode sputtered on the FTO substrate surface

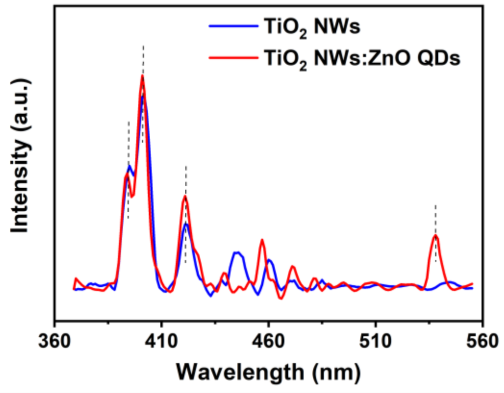

Fig. S3 PL spectrum of the TiO_2 NWs and TiO_2 NWs: ZnO QDs film

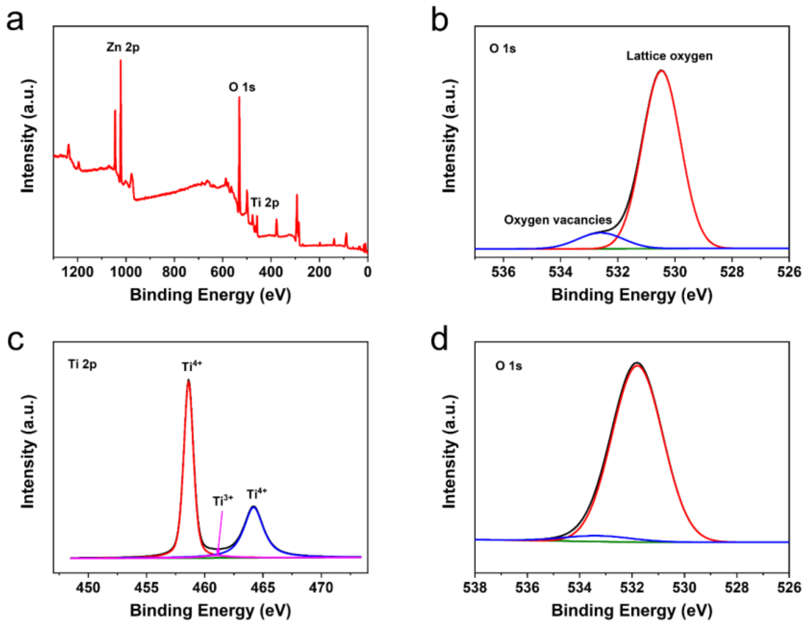

Fig. S4 a XPS spectra of the TiO_2 NWs: ZnO QDs film. **b** O 1s spectra of the TiO_2 NWs: ZnO QDs film. **c** Ti 2p spectra of the pure TiO_2 NWs film. **d** O 1s spectra of the pure TiO_2 NWs film

Mechanism

To study the carrier transport mechanism of the device, the I-V curve of the device is fitted under different theories, including Fowler-Nord heim tunneling, Schottky emission, and Poole-Frankel emission [S1-S3]. As shown in Fig. S5, it can be observed that the *I-V* curve does not exhibit linearity under the Poole-Frankel emission and Fowler-Nord heim tunneling model. However, Schottky emission at low bias voltages demonstrates some degree of linearity. This may be attributed to the dominance of the Schottky barrier between the electrode and the resistive active layer for electron transport at low voltages. Furthermore, the I-V curve is fitted to the Space-Charge Limited Current (SCLC) model, as shown in Fig. S6. It can be seen that, compared to other mechanisms, the carrier transport behavior is more similar to the behavior in SCLC. Fig. S6a depicts the fitting results of the *I–V* curve in the positive region. When a low voltage is applied to the device, the slope of the curve is 0.65, corresponding to ohmic conduction ($I \propto V$). The fitted slope is less than 1, which can be attributed to the influence of the Schottky emission conduction of carriers at low bias voltages. The slope rises to 2.33 ($I \propto V^2$) with the increase in voltage and then increases to 8.61 ($I \propto V^n$) at high voltage. The fitting results demonstrate that the SCLC model dominates the carrier transport mechanism [S4, S5]. Furthermore, the fitting results in the negative region (from 0.79 to 1.82, and finally to 3.51) demonstrate transport behavior (SCLC) similar to the positive region, as shown in Fig. S6b.

Furthermore, based on the fitting results, the resistive switching mechanism of the device is further elucidated using energy band alignment diagrams and a physical model. Oxygen defects on the surface of ZnO QDs act as trapping/de-trapping centers during the resistive switching process, while TiO_2 NWs serve as the charge transport layer. Fig. S7a illustrates the initial state energy band alignment diagram of the device. When a voltage is applied, electrons pass over the Schottky barrier from the FTO bottom electrode to the defects in the surface ZnO QDs, as shown in Fig. S7b. With an increase in applied voltage, more electrons fill the defects, leading to an increase in device conductivity. These trapped electrons undergo slow release spontaneously, resulting in the relaxation decay of device conductivity and PPF behavior. Furthermore, as the number of applied sweeping voltage increases, the trap centers gradually become filled. At this point, the injected electrons can move freely, creating a conductive path between the top and bottom electrodes. Therefore, the conductivity changes slowly, tending to saturate, as shown in Fig. S7c. Conversely, under a negative voltage, the electrons are gradually de-trapped from the trapping centers to the FTO bottom electrode, thus causing a decrease in the device conductance, as shown in Fig. S7d.

It is worth noting that the active Ag electrode introduces considerations regarding the oxidation and migration of the metal under an electrical field. However, Ag migration relies on oxidation reactions occurring with defects in the bulk film, which results in the typical non-volatile characteristics of the conductance [S6]. This is inconsistent with the results observed in Fig. S14. Furthermore, the migration of active metals in oxide films critically depends on defects within the film [S7]. The presence of Ti interstitial

defects and oxygen vacancies promotes the migration of Ag^+ ions in the TiO_2 dielectric layer, leading to the migration of Ag^+ ions. As shown in Fig. S4d, only a small amount of Ti^{3+} and oxygen vacancies are observed in pure TiO_2 NWs films, limiting the migration of Ag^+ ions. For the resistive switching active layer with a thickness in the micrometer range, the contribution of Ag diffusion length in bulk films to the resistive switching under a small electric field intensity is minimal [S8]. Therefore, the Ag migration is challenging in generating the resistive switching mechanism of the device.

The light-induced response of the ATZ-based device can be attributed to the photoconductive effects of TiO_2 NWs and ZnO QDs [S9, S10]. The electron-hole pairs are excited and separated under UV radiation owing to the large photon energy, as illustrated in Fig. S8a-(I). The photo-generated holes are retained at the bottom of the valence band, while the photo-generated electrons are excited and move to the conduction band, thereby increasing the conductance of the device. Furthermore, the carrier transport behavior occurring at the TiO_2/ZnO interface effectively enhances the light-induced response and persistent photoconductivity effect [S11], as shown in Fig. S8a-(II). Due to the abundant defects on the surface of ZnO QDs, photo-generated electrons in TiO_2 can easily transfer to ZnO, further promoting the excitation of photo-generated electrons in TiO_2. The physical model depicted in Fig. S8a-(III) provides an intuitive representation of the effective separation of carriers at the TiO_2/ZnO interface. Upon closing off the UV light, the photo-generated electron-hole pairs excited in TiO_2 undergo slow recombination, resulting in a gradual decrease in conductance, as depicted in Fig. S8b-(I). Notably, the effective lifetime of photo-generated electrons captured in ZnO is extended as they need to overcome the interface barrier for release (Fig. S8b-(II)), leading to an increased relaxation time. This slow-decaying light response contributes to the persistent photoconductivity effect. Furthermore, when multiple light pulse irradiations are applied to the device, the photo-generated electrons initially excited cannot recombine with holes within a limited timeframe, leading to the accumulation of photo-generated electrons under subsequent light pulse excitation. Therefore, the light response from the second light pulse is significantly greater than the first, facilitating the implementation of the light-induced PPF behavior. A physical model, as presented in Fig. S8b-(III), is shown to provide an intuitive representation of the recombination process of photo-generated electron-hole pairs at the TiO_2/ZnO interface.

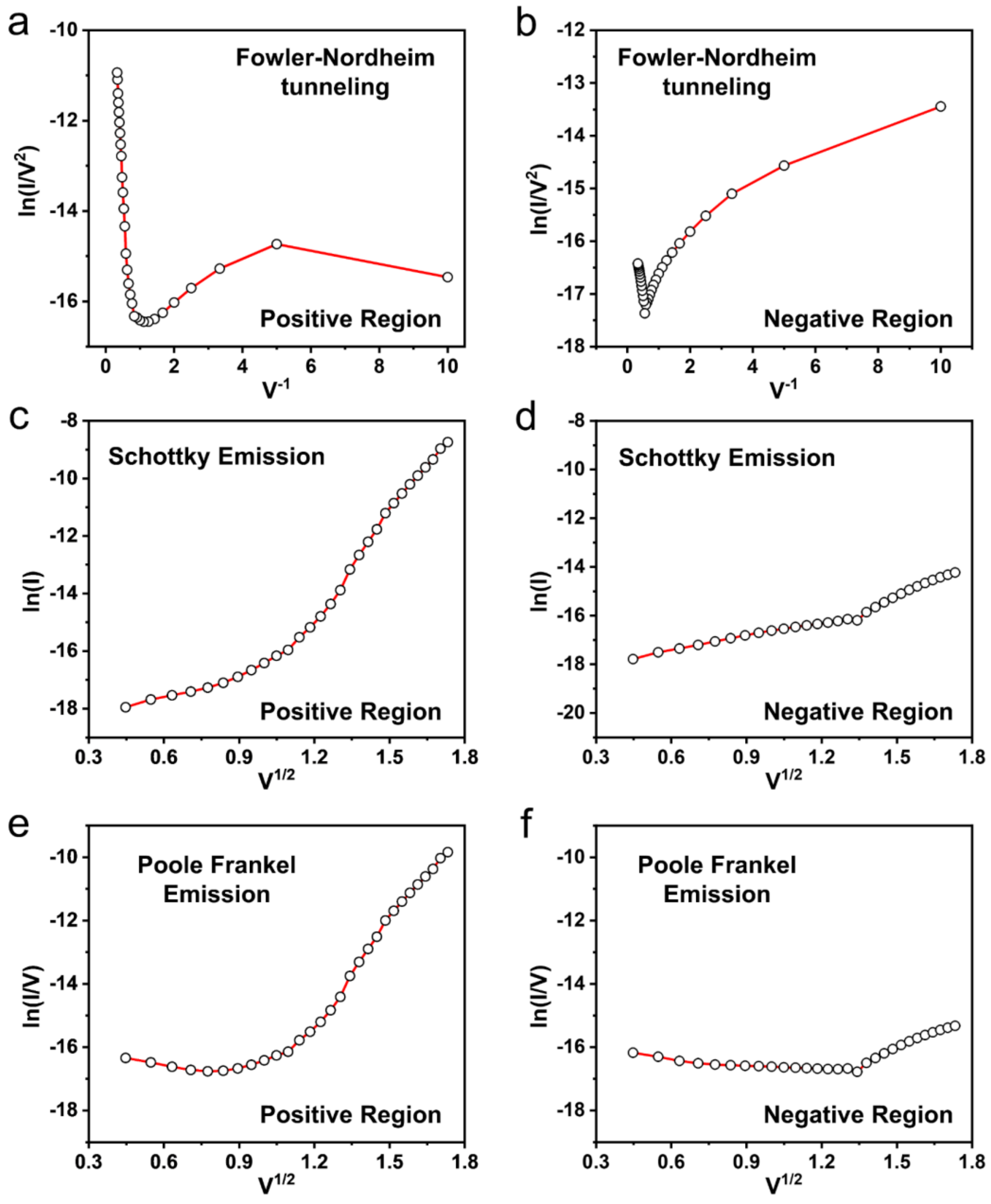

Fig. S5 Ln(I/V^2)–V^{-1} plots (Fowler-Nordheim tunneling) of the I–V characteristic curve for the ATZ-based device under **a** positive region and **b** negative region. Ln(I)–$V^{1/2}$ plots (Schottky emission) of the I–V characteristic curve under the **c** positive region and **d** negative region. Ln(I/V)–$V^{1/2}$ plots (Poole Frankel emission) of the I–V characteristic curve under **e** positive region and **f** negative region.

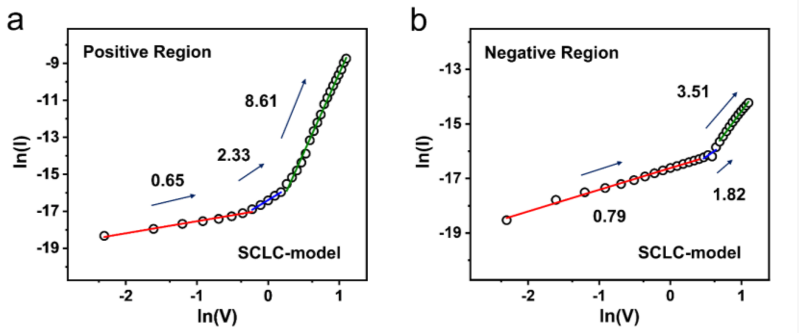

Fig. S6 Ln–ln plots of the *I*–*V* characteristic curve for the ATZ-based device under **a** positive region and **b** negative region

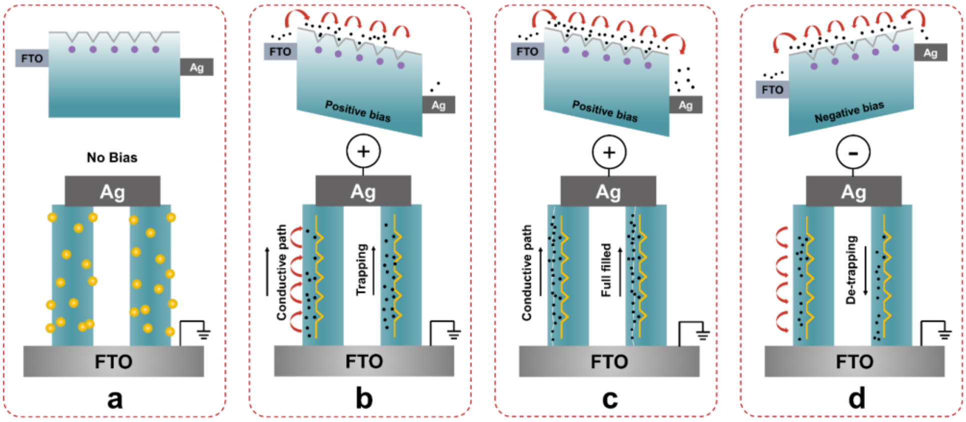

Fig. S7 Energy band alignment diagrams and resistive switching physical model of the device under the **a** initial state, **b** positive bias, **c** sufficient positive bias, and **d** negative bias

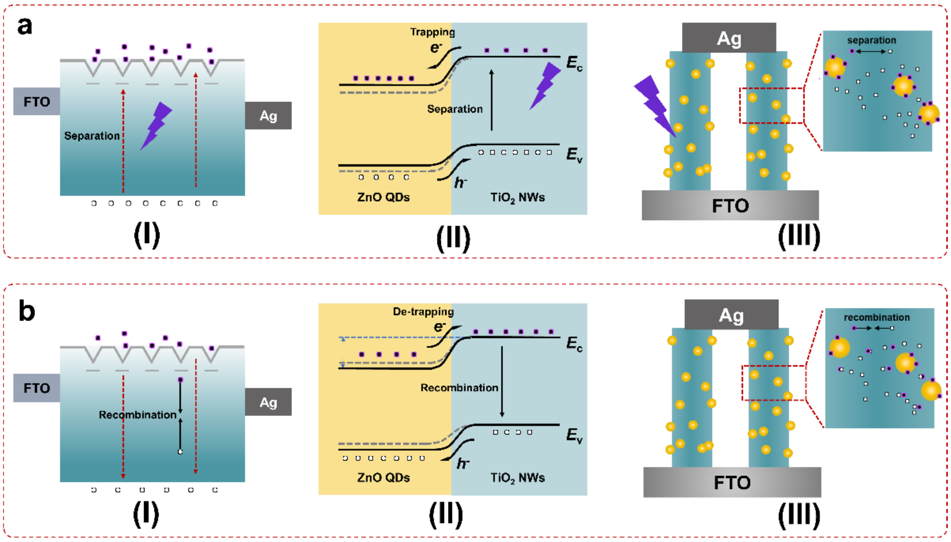

Fig. S8 **a** Under light illumination, (I) the energy band diagram of the device, (II) the energy band diagram of the TiO_2/ZnO interface, and (III) the schematic of the physical model. **b** Under the dark condition, (I) the energy band diagram of the device, (II) the

energy band diagram of the TiO_2/ZnO interface, and (III) the schematic of the physical model.

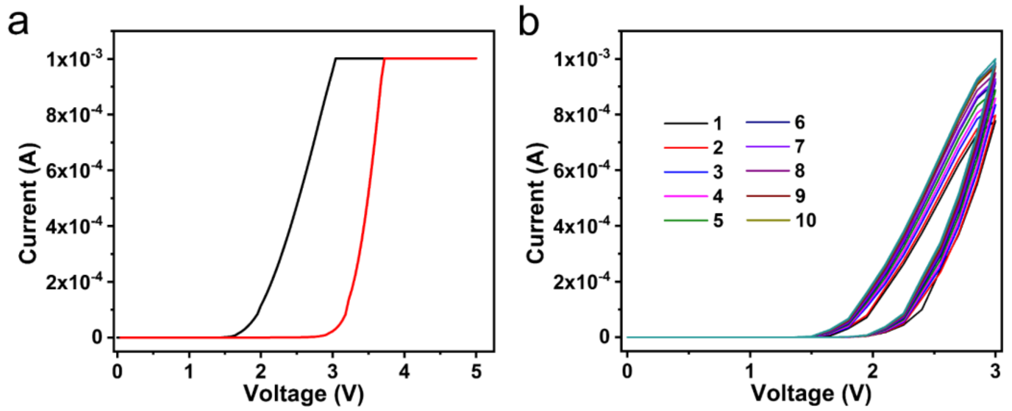

Fig. S9 a *I-V* curve of the device at large forming voltage. **b** Continuous I-V curves of the device after the forming process

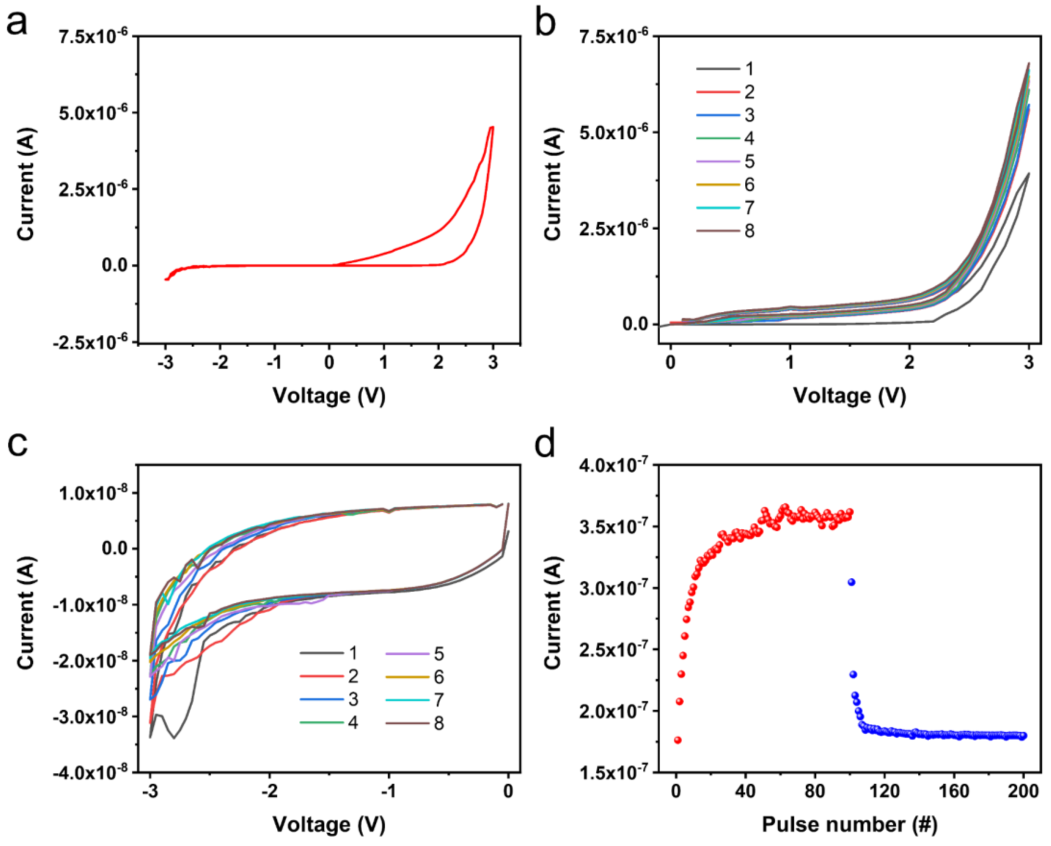

Fig. S10 a I-V curve of the pure TiO_2 NWs-based device. Consecutive I–V curves of the pure TiO_2 NWs-based device for 8 cycles under **b** positive sweeping voltage and **c** negative sweeping voltage. **d** LTP/LTD properties of the pure TiO_2 NWs-based device (+3 V for potentiation, –3 V for depression)

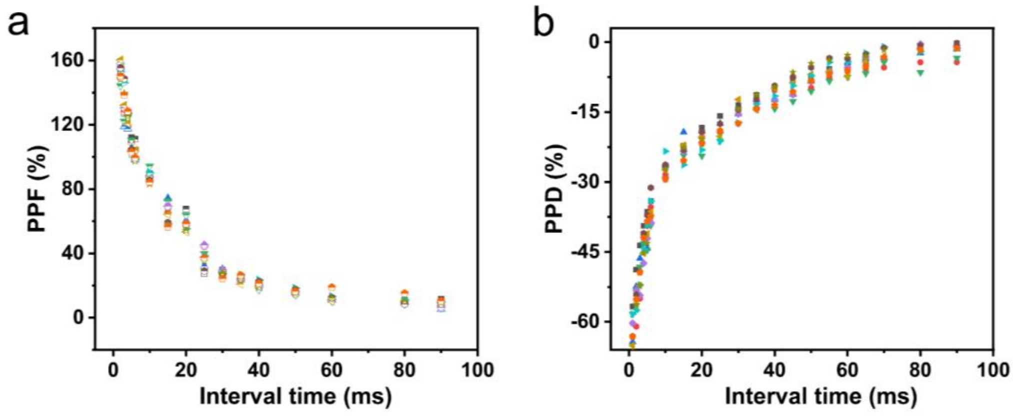

Fig. S11 a Electric-induced PPF property of the device for 10 cycles. **b** Electric-induced PPD property of the device for 10 cycles

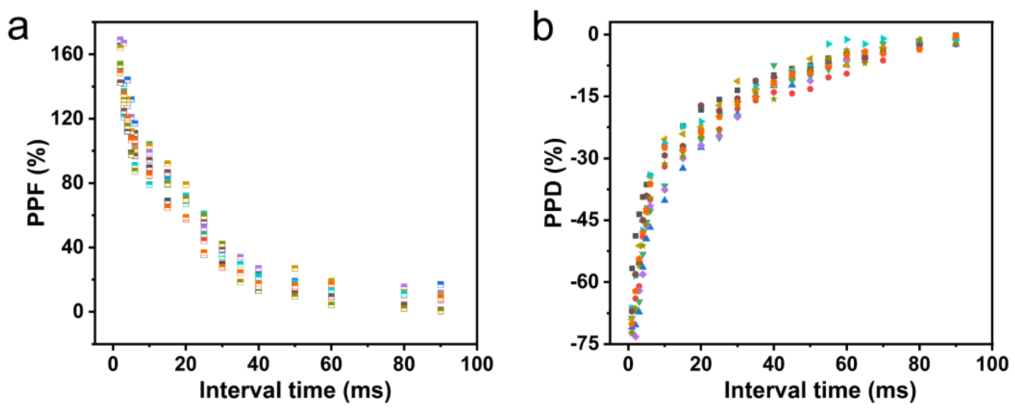

Fig. S12 a Electric-induced PPF property for 10 devices. **b** Electric-induced PPD property for 10 devices

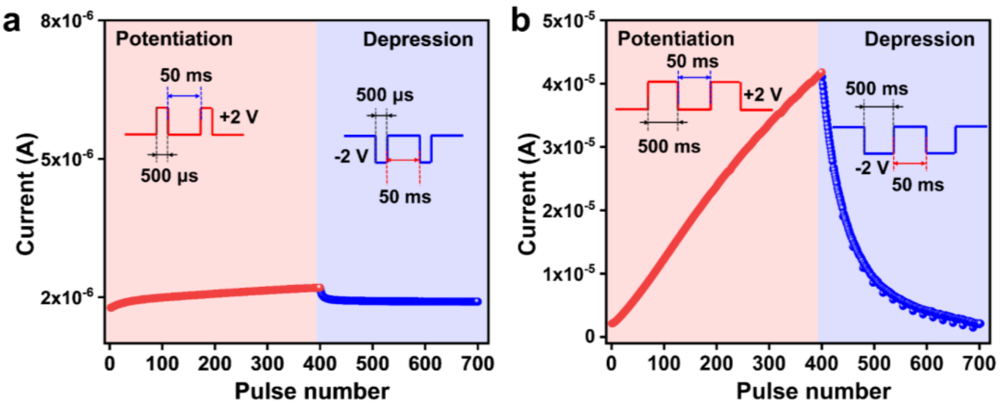

Fig. S13 a LTP/LTD behavior at the pulse width of 500 μs. **b** LTP/LTD behavior at the pulse width of 500 ms

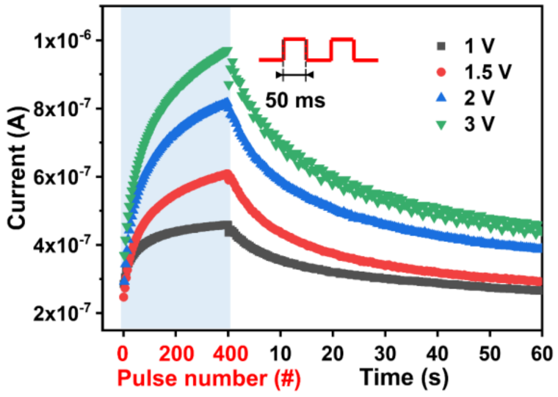

Fig. S14 Spontaneous decay behavior of the device after being excited by 1 V, 1.5 V, 2 V, and 3 V of positive voltage pulses (reading voltage: 0.1 V).

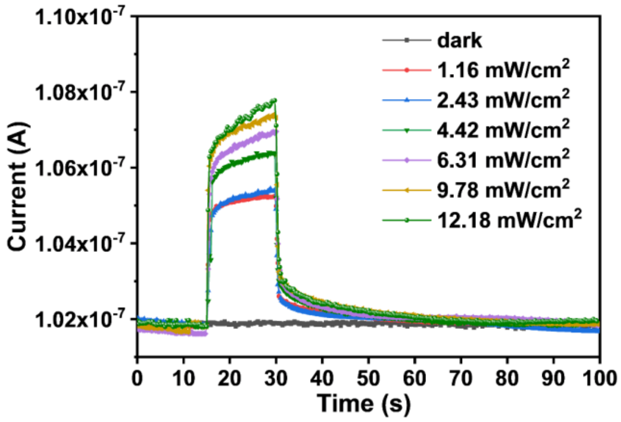

Fig. S15 Current responses of the pure TiO_2 NWs-based device under different light densities (dark, 1.16, 2.43, 4.42, 6.31, 9.78, and 12.18 mW/cm^2)

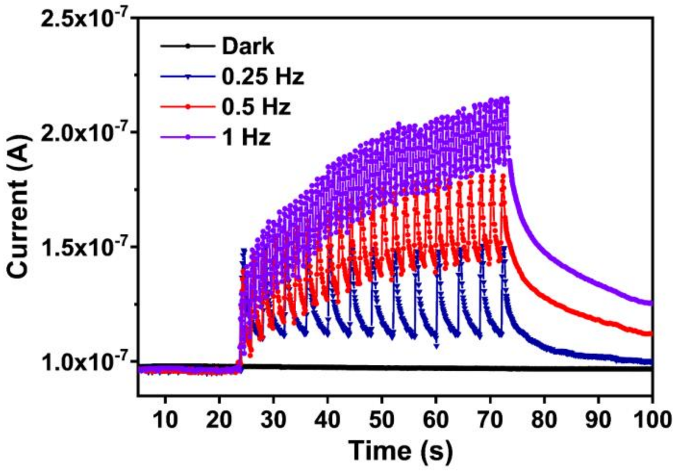

Fig. S16 Current responses of the ATZ-based device under different light frequencies (dark, 0.25, 0.5, and 1 Hz)

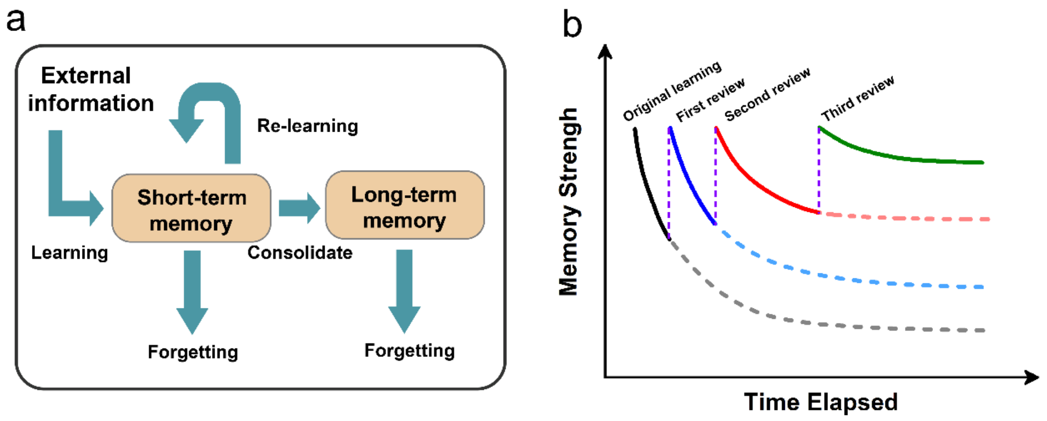

Fig. S17 a Schematic of the learning-forgetting-relearning behavior. **b** Ebbinghaus forgetting curve model

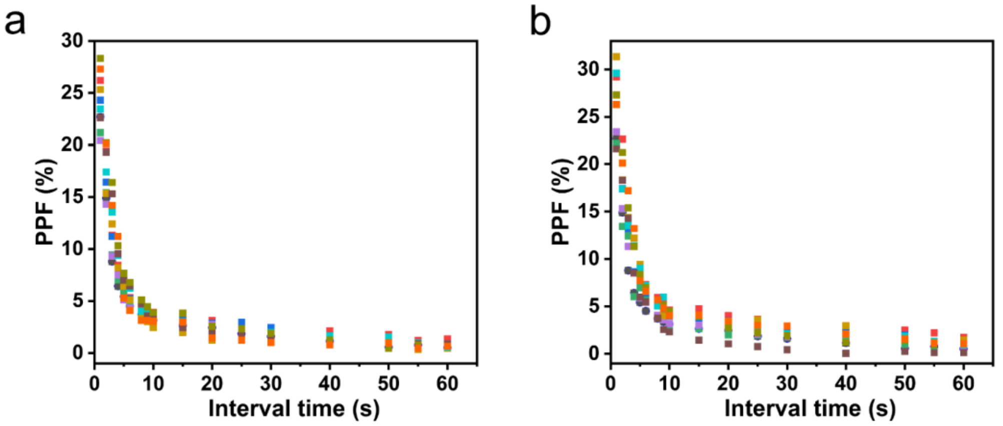

Fig. S18 a Light-induced PPF property of the device for 10 cycles. **b** light-induced PPF property for 10 devices

Weight update model

The synaptic plasticity of the device is used to implement the simulation of the Artificial Neural Network (ANN). The prevalent "vector-matrix" multiplication operation in neural network computations can be efficiently executed on the memristor array using Kirchhoff's Law. The matrix function can be expressed as follows:

$$\begin{bmatrix} I_1 \\ I_2 \\ \vdots \\ I_i \end{bmatrix} = \begin{bmatrix} G_{1,1} & G_{1,2} \cdots & G_{1,j} \\ G_{2,1} & G_{2,2} \cdots & G_{2,j} \\ & \vdots & \\ G_{i,1} & G_{i,2} \cdots & G_{i,j} \end{bmatrix} \square \begin{bmatrix} V_1 \\ V_2 \\ \vdots \\ V_j \end{bmatrix} \tag{S1}$$

where V_j represents the voltage vector, and I_j denotes the output current vector. Applying V_j to the word lines of the array allows the computation results from I_j to be collected along each bit line. The I_j can be expressed as follows:

$$I_i = \sum_{n=1}^{j} G_{i,n} \cdot V_n = G_{i,1} \cdot V_1 + G_{i,2} \cdot V_2 + \cdots + G_{i,j} \cdot V_j \tag{S2}$$

A fully connected ANN was constructed, comprising 784 input neurons, 100 hidden neurons, and 10 output neurons, to implement neuromorphic computing for the classification of the modified MNIST handwritten digit dataset. In this network, neurons in each layer are interconnected through synapses, with the connection strength representing the weights. Before training, 28 × 28-pixel handwritten digit images were converted to grayscale values and initialized as inputs for the 784 neurons in the input layer. The training process comprises forward propagation, loss computation, backpropagation, and weight updates [S12]. During forward propagation, the loss is calculated between the output and target signals. Subsequently, backpropagation is employed to adjust the connection weights among neurons across various layers. The update rate of weights is set to 0.1 times the estimated weight error each time, meaning a learning rate of 0.1, and the weight adjustments are iteratively executed until the loss converges to the target value. The LTP/LTD characteristics of the device were employed for the matrix multiplication in the neural network to compute weight updates. A mathematical model was established to update the synaptic weights in neural network computing. The equations are as given below:

$$G_{\mathrm{LTP}} = B \cdot (1 - e^{\frac{-P}{A}}) + G_{\min}, \tag{S3}$$

$$G_{\mathrm{LTD}} = G_{\max} - B \cdot (1 - e^{\frac{(P_{\max} - P)}{A}}) + G_{\min}, \tag{S4}$$

$$B = \frac{G_{\max} - G_{\min}}{1 - e^{\frac{-P_{\max}}{A}}}, \tag{S5}$$

$$\mathrm{ANL} = \frac{G_{\mathrm{LTP}}(\frac{P}{2}) - G_{\mathrm{LTD}}(\frac{P}{2})}{G_{\max} - G_{\min}}, \tag{S6}$$

where G_{LTP} and G_{LTD} represent the conductance values of the experimental LTP and LTD, respectively; $G_{\max}$ and $G_{\min}$ represent the maximum and minimum conductance values, respectively; P denotes the pulse number during the operation of the device from $G_{\max}$ to $G_{\min}$; $\mathrm{P}_{\max}$ denotes the maximum number of pulses; A denotes the parameter corresponding to the nonlinearity of the weight update; B denotes a function of A; and ANL represents the asymmetric nonlinearity factor between LTP and LTD.

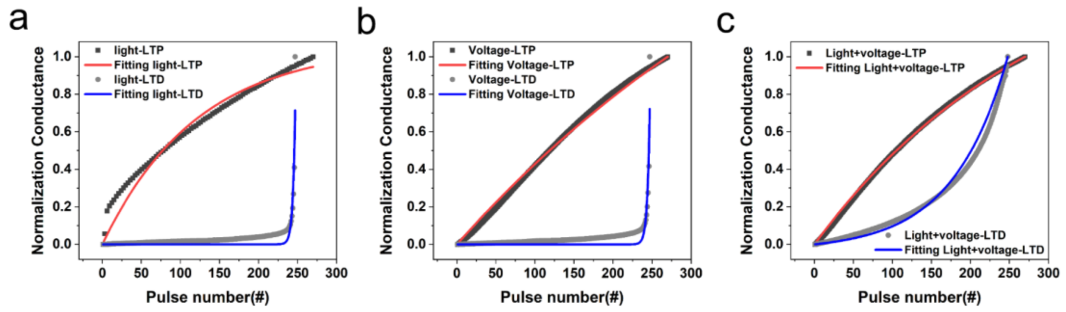

Fig. S19 Normalized LTP/LTD behaviors and fitting curves under **a** pure light, **b** pure voltage, and **c** light plus voltage

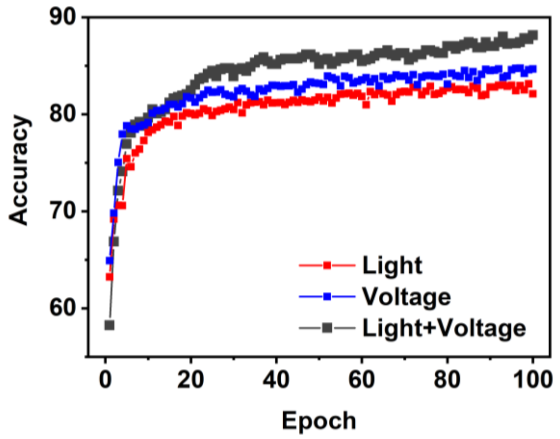

Fig. S20 Variation in accuracy during 100 training epochs under pure light, pure voltage, and light plus voltage conditions

Table S1 Comparison of nonlinearity and asymmetry parameters

Parameter	Nonlinearity		ANL
	LTP	LTD	
Light	3.65	6.65	0.7119
Voltage	0.91	7.49	0.5605
Light + Voltage	1.69	4.76	0.4337

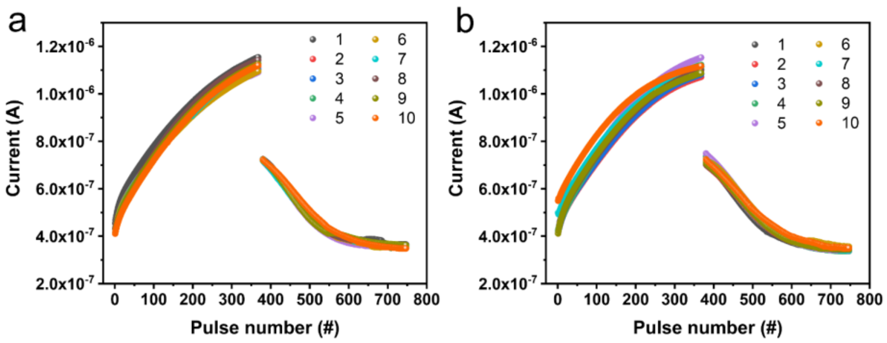

Fig. S21 **a** Cycle-to-cycle and **b** device-to-device performance of LTP/LTD properties (light plus voltage)

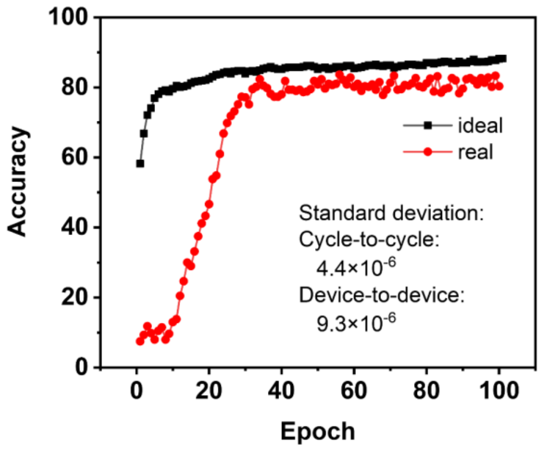

Fig. S22 Recognition accuracy of ideal memristor and non-ideal memristor during 100 training epochs

Table S2 Comparisons of the performance of several synaptic devices

Device structure	Conductance pulses	Weight increase pluses	Weight decrease pluses	Non-linearity [LTP/D]	Accuracy	Light-induced synaptic plasticity	Classical conditioning	Refs.
Pt/BTO/SNTO	200	1.3 V/50 ns	-1.75 V/50 ns	0.25/0.25	96.4%	-	-	[S13]
Pt/WO_{3-x}/W	200	2 V/10 ms	0.2 V/10 ms	-	-	-	Yes	[S14]
Au/$CsCu_2I_3$/ITO	60	-2 V/7 ms	2 V/10 ms	1.8/1.3	84.5%	-	-	[S15]
Multilayer BP device	400	3 mW/cm^2 light/20 ms	6.8 mW/cm^2 light/20 ms	-	90%	LTP/LTD, STM/LTM	-	[S16]
Ag/ZnO/ITO	100	0.5 V/0.1 s	-0.5 V/0.1 s	-	-	LTP/D, PPF/D	-	[S6]
ITO/CQDs/Au	-	2.4 V/<500 ns	-2.4 V/<500 ns	-	96.7%	-	Yes	[S17]
Ag/Nb: $SrTiO_3$/ITO	-	10-30 mW/cm^2 light/0.05-1 Hz	-0.15 V/0.5 s	-	-	LTP/D, PPF/D, LTM/STM, learning--forgetting-relearning		[S18]
ITO/ZnO/Ag	50	+2 V/10 ms	-2 V/10 ms	-	86.7%	LTP/D, PPF/D	-	[S19]
Au/ion gel/GO/IGZO	100	-1 V/50 ms	1 V/50 ms	4.24/8	62%	LTP/D, PPF/D	-	[S20]

Au/Spiro-OMeTAD/PFEP/ITO	40	1 V/10 ms	-1 V/10 ms	-	-	LTP/D, PPF/D, PTP/D, SRDP	Yes	[S21]
Ag/TiO_2 NWs: ZnO QDs/FTO	700	+2 V/50 ms	-2 V/50 ms	1.69/4.76	88.9%	LTP/D, PPF/D, LTM/STM, learning-forgetting-relearning	Yes	This work

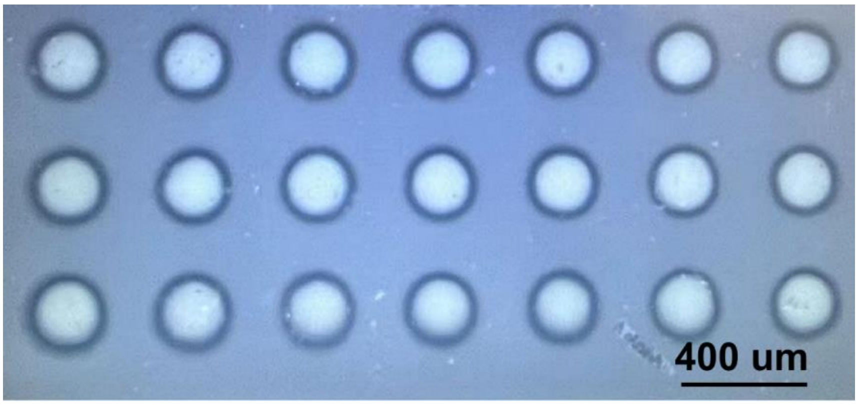

Fig. S23 Optical image of the 3 × 7 memristive array

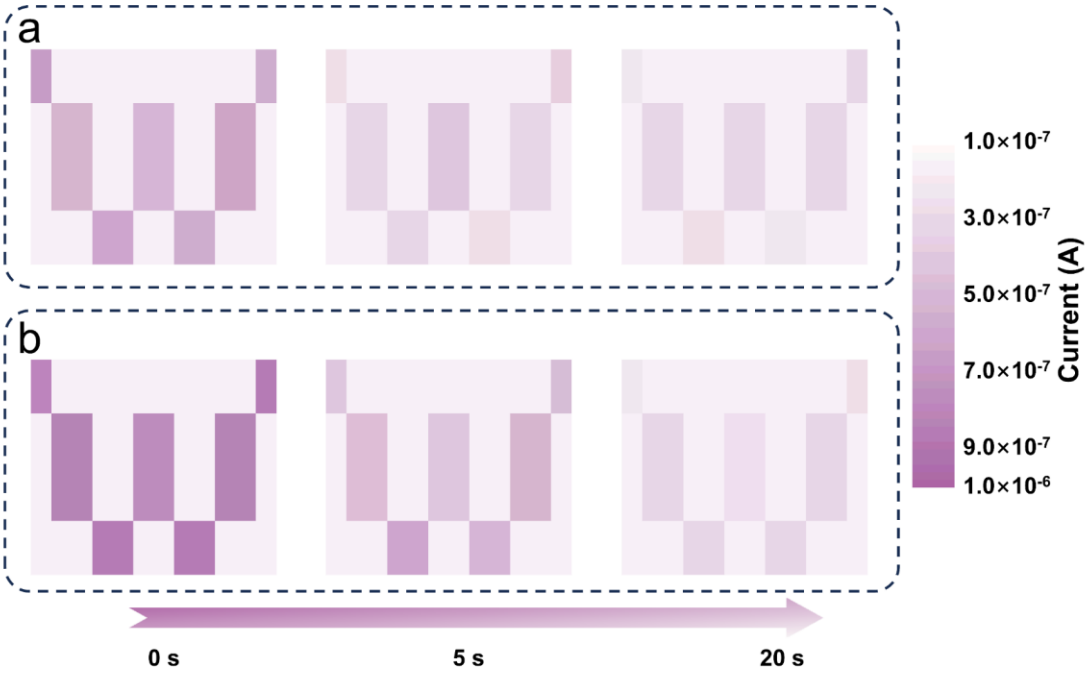

Fig. S24 a The variation in conductance-mapped pattern (letter "W") with decay time (0, 5, and 20 s) after being excited by 200 electrical pulses (width: 50 ms, interval: 50 ms). **b** The variation in conductance-mapped pattern (letter "W") with decay time (0, 5, and 20 s) after excited by 200 electrical pulses (width: 50 ms, interval: 50 ms) plus light pulse (light density: 3.86 mW/cm^2, duration: 2 s)

Supplementary References

[S1] M. Yang, H. Wang, X. H. Ma, H. X. Gao, B. Wang, Effect of nitrogen-accommodation ability of electrodes in SiN_x-based resistive switching devices. Appl. Phys. Lett. **111**, 233510 (2017). https://doi.org/10.1063/1.4996618

[S2] Y. F. Chang, B. Fowler, Y. C. Chen, Y. T. Chen, Y. Z. Wang et al. , Intrinsic SiO_x-based unipolar resistive switching memory. II. Thermal effects on charge transport and characterization of multilevel programing. J. Appl. Phys. **116**, 043709 (2014). https://doi.org/10.1063/1.4891244

[S3] G. Zhao, Y. L. Yin, Y. H. Peng, W. J. Yang, Y. H. Liu et al. , Effect of hydrogen ions in the adsorbed water layer on the resistive switching properties of hexagonal WO_3 nanowire. J. Appl. Phys. **126**, 054303 (2019). https://doi.org/10.1063/1.5093277

[S4] S. Ge, L. Huang, C. Pan, Halide perovskite single crystals for resistive switching. Sci. Bull. **67**, 1018 (2022). https://doi.org/10.1016/j.scib.2022.02.001

[S5] D. Bhatnagar, A. Kumar, K. Prabahar, M. Suri, A. Srinivas et al. , Non-volatile resistive switching in oxide ion conductor $BiYO_3$ thin films. Appl. Phys. Lett. **113**, 162101 (2018). https://doi.org/10.1063/1.5041790

[S6] P. S. Subin, P. S. Midhun, A. Antony, K. J. Saji, M. K. Jayaraj, Optoelectronic synaptic mimicked in ZnO-based artificial synapse for neuromorphic image sensing application. Mater. Today Commun. **33**, 104232 (2022). https://doi.org/10.1016/j.mtcomm.2022.104232

[S7] M. S. Irshad, A. Abbas, H. H. Qazi, M. H. Aziz, M. Shah et al. , Role of point defects in hybrid phase TiO_2 for resistive random-access memory (RRAM), Mater. Res. Express **6**, 076311 (2019). https://doi.org/10.1088/2053-1591/ab17b5

[S8] C. P. Veeramalai, P. Kollu, G. C. Lin, X. M. Zhang, C. A. B. Li, Fabrication of graphene: CdSe quantum dots/CdS nanorod heterojunction photodetector and role of graphene to enhance the photoresponsive characteristics. Nanotechnology **32**, 315204 (2021). https://doi.org/10.1088/1361-6528/abf87a

[S9] M. Kumar, J. Y. Park, H. Seo, Boosting self-powered ultraviolet photoresponse of TiO_2-based heterostructure by flexo-phototronic effects. Adv. Opt. Mater. **10**, 2102532 (2022). https://doi.org/10.1002/adom.202102532

[S10] M. Gong, Q. Liu, B. Cook, B. Kattel, T. Wang, et al., All-printable ZnO quantum dots/graphene van der waals heterostructures for ultrasensitive detection of ultraviolet light. ACS Nano **11**, 4114 (2017). https://doi.org/10.1021/acsnano.7b00805

[S11] V. Klee, E. Preciado, D. Barroso, A. E. Nguyen, C. Lee, et al., Superlinear composition-dependent photocurrent in CVD-grown monolayer $MoS_{2(1-x)}Se_{2x}$ alloy devices. Nano Lett. **15**, 2612 (2015).

https://doi.org/10.1021/acs.nanolett.5b00190

[S12] X. Y. Wang, P. Xie, B. H. Chen, X. C. Zhang, Chip-based high-dimensional optical neural network. Nano-Micro Lett. **14**, 221 (2022). https://doi.org/10.1007/s40820-022-00957-8

[S13] J. K. Li, C. Ge, J. Y. Du, C. Wang, G. Z. Yang, K. J. Jin, Reproducible ultrathin ferroelectric domain switching for high-performance neuromorphic computing. Adv. Mater. **32**, 1905764 (2019). https://doi.org/10.1002/adma.201905764

[S14] Y. Lin, F. Q. Meng, T. Zeng, Q. H. Zhang, Z. Q. Wang et al. , Direct observation of oxygen ion dynamics in a WO_{3-x} based second-order memristor with dendritic integration functions. Adv. Funct. Mater. **33**, 2302787 (2023). https://doi.org/10.1002/adfm.202302787

[S15] K. J. Kwak, J. H. Baek, D. Lee, I. H. Im, J. Kim et al. , Ambient stable all inorganic $CsCu_2I_3$ artificial synapses for neurocomputing. Nano Lett. **22**, 6010 (2022). https://doi.org/10.1021/acs.nanolett.2c01272

[S16] T. Ahmed, M. Tahir, M. X. Low, Y. Y. Ren, S. A. Tawfik et al. , Fully light-controlled memory and neuromorphic computation layered black phosphorus. Adv. Mater. **33**, 2004207 (2020). https://doi.org/10.1002/adma.202004207

[S17] T. Q. Yu, Y. Fang, X. Y. Chen, M. Liu, D. Wang et al. , Hybridization state transition-driven carbon quantum dot (CQD)-based resistive switches for bionic synapses. Mater. Horizons **10**, 2181 (2023). https://doi.org/10.1039/D3MH00117B

[S18] S. Gao, G. Liu, H. L. Yang, C. Hu, Q. L. Chen et al. , An oxide schottky junction artificial optoelectronic synapse, ACS Nano **13**, 2634 (2019). https://doi.org/10.1021/acsnano.9b00340

[S19] T. Y. Wang, J. L. Meng, Q. X. Li, Z. Y. He, H. Zhu et al. , Reconfigurable optoelectronic memristor for in-sensor computing applications, Nano Energy **89**, 106291 (2021). https://doi.org/10.1016/j.nanoen.2021.106291

[S20] J. Sun, S. Oh, Y. Choi, S. Seo, M. J. Oh et al. , Optoelectronic synapse based on IGZO-alkylated graphene oxide hybrid structure, Adv. Funct. Mater. **28**, 1804397 (2018). https://doi.org/10.1002/adfm.201804397

[S21] S. S. Han, T. Ma, H. Li, J. L. Wu, R. Liu et al. , Photoferroelectric perovskite synapses for neuromorphic computing, Adv. Funct. Mater. 2309910 (2023). https://doi.org/10.1002/adfm.202309910
